# Beta cell function in participants with single or multiple islet autoantibodies at baseline in the TEDDY Family Prevention Study: TEFA

**DOI:** 10.1002/edm2.198

**Published:** 2020-11-05

**Authors:** Maria Månsson Martinez, Falastin Salami, Helena Elding Larsson, Jorma Toppari, Åke Lernmark, Jukka Kero, Riitta Veijola, Jaakko J Koskenniemi, Päivi Tossavainen, Markus Lundgren, Henrik Borg, Anastasia Katsarou, Marlena Maziarz, Carina Törn, The TEDDY Family (TEFA) Study Group

**Affiliations:** ^1^ Department of Clinical Sciences Lund University/CRC Skåne University Hospital Malmö Sweden; ^2^ Department of Pediatrics Turku University Hospital Turku Finland; ^3^ Institute of Biomedicine Research Centre for Integrative Physiology and Pharmacology and Research Centre for Population Health University of Turku Turku Finland; ^4^ Department of Pediatrics PEDEGO Research Unit MRC Oulu University of Oulu and Oulu University Hospital Oulu Finland

**Keywords:** HbA1c, intravenous glucose tolerance test, oral glucose tolerance test

## Abstract

**Aim:**

The aim of the present study was to assess beta cell function based on an oral glucose tolerance test (OGTT) in participants with single islet autoantibody or an intravenous glucose tolerance test (IvGTT) in participants with multiple islet autoantibodies.

**Materials and methods:**

Healthy participants in Sweden and Finland, between 2 and 49.99 years of age previously identified as positive for a single (n = 30) autoantibody to either insulin, glutamic acid decarboxylase, islet antigen‐2, zinc transporter 8 or islet cell antibodies or multiple autoantibodies (n = 46), were included. Participants positive for a single autoantibody underwent a 6‐point OGTT while participants positive for multiple autoantibodies underwent an IvGTT. Glucose, insulin and C‐peptide were measured from OGTT and IvGTT samples.

**Results:**

All participants positive for a single autoantibody had a normal glucose tolerance test with 120 minutes glucose below 7.70 mmol/L and HbA1c values within the normal range (<42 mmol/mol). Insulin responses to the glucose challenge on OGTT ranged between 13.0 and 143 mIU/L after 120 minutes with C‐peptide values between 0.74 and 4.60 nmol/L. In Swedish participants, the first‐phase insulin response (FPIR) on IvGTT was lower in those positive for three or more autoantibodies (n = 13; median 83.0 mIU/L; range 20.0‐343) compared to those with two autoantibodies (n = 15; median 146 mIU/L; range 19.0‐545; *P* = .0330).

**Conclusion:**

Participants positive for a single autoantibody appeared to have a normal beta cell function. Participants positive for three or more autoantibodies had a lower FPIR as compared to participants with two autoantibodies, supporting the view that their beta cell function had deteriorated.

## Graphical abstract

The aim was to assess beta cell function based on an oral glucose tolerance test (OGTT) in participants with single islet autoantibody or an intravenous glucose tolerance test (IvGTT) in participants with multiple islet autoantibodies. Participants positive for a single autoantibody appeared to have a normal beta cell function. Participants positive for three or more autoantibodies had a lower FPIR as compared to participants with two autoantibodies, supporting the view that their beta cell function had deteriorated.

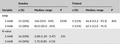

## 1 INTRODUCTION

Islet autoantibodies against either glutamic acid decarboxylase autoantibodies (GADA), islet antigen‐2 autoantibodies (IA‐2A), insulin autoantibodies (IAA) or zinc transporter‐8 autoantibodies (ZnT8A), alone or in combination, are strong biomarkers of ongoing islet autoimmunity and define the first step towards clinical onset of type 1 diabetes. The second step is an impaired beta cell function that can be identified and monitored by oral (OGTT) or intravenous (IvGTT) glucose tolerance tests where glucose, insulin and C‐peptide are measured. Glucose values > 7.80‐11.0 mmol/L at 120 minutes in OGTT indicate impaired glucose tolerance (IGT). A loss of the first‐phase insulin response (FPIR) in the IvGTT is an early indication of diminished beta cell function followed by a reduced secondary insulin response at a later stage in the disease process.^1^, ^2^ Increased fasting glucose values are relatively late indicators of declining beta cell function in children, while more important as early indicators in adults.^3^ Metabolic derangement can also be assessed by measurement of HbA1c, which reflects the glucose levels over the last two to three months. HbA1c can be a useful biomarker, if it is regularly measured, to monitor the progression if the appearance of the first autoantibody to the clinical onset of type 1 diabetes is lengthy.^4^

The finding of a single autoantibody when participants at risk are screened usually indicates a 10%‐15% risk to progress to clinical diabetes over the next five to ten years. Multiple (two or more) autoantibodies indicate that more than 70% may progress to clinical onset within ten years.^5^, ^6^

The primary objective of the TEDDY Family Study (TEFA) is to evaluate the effect of a gluten free diet (GFD) over 18 months on beta cell function and glucose metabolism in a prospective randomized trial.

In the present baseline study, the aim was to assess beta cell function based on an oral glucose tolerance test (OGTT) in participants with single islet autoantibody or an intravenous glucose tolerance test (IvGTT) in participants with multiple islet autoantibodies. Another aim was to compare participants with two different autoantibodies to those with three or more.

## 2 MATERIALS AND METHODS

### 2.1 Study population

Participants eligible for the TEFA study were selected from a population of autoantibody‐positive first‐degree relatives of participants in The Environmental Determinants of Diabetes (TEDDY) study or those identified as autoantibody positive in one of the following studies: The Diabetes Prediction in Skåne (DiPiS) study, the Type 1 Diabetes Prediction and Prevention (DIPP) study in Finland or in TrialNet screening. The major inclusion criteria for the TEFA randomized trial were as follows: (a) participants were between 2 and 49.99 years of age, (b) participants were persistently positive (positive in at least two consecutive visits) for one islet autoantibody (GADA, IA‐2A, IAA or ZnT8R/W/QA) and had impaired glucose metabolism or were persistently positive for at least two autoantibodies. The exclusion criteria were ongoing immunosuppressant therapy (topical or inhaled steroids were accepted), diabetes, treatment with any anti‐diabetic medications, clinically relevant abnormal haematology results at screening, participation in other clinical drug trials within the previous three months, history of hypercalcemia, presence of associated serious disease or diabetes‐protective DQB1*06:02 allele.

Prior to inclusion into the randomized trial, potential participants were tested with either an OGTT in the case of positivity for a single autoantibody or an IvGTT if positive for multiple autoantibodies. Data from these two visits are presented in the current study. Participants were eligible to the randomized trial if they had a single autoantibody combined with impaired glucose tolerance (IGT) based on the outcome from OGTT. Participants with multiple autoantibodies (two or more) qualified for inclusion irrespective of the glycemic status. All study participants signed an informed consent. The study was approved by the Regional Ethical Committee in Lund, Sweden and by the Ethics Committee of the Hospital District of Southwest Finland in Turku, Finland.

### 2.2 Assessment of beta cell function

In Sweden, a total of 3738 family members and in Finland 1526 family members of participants enrolled in the TEDDY study were screened for autoantibodies.

Participants positive for a single autoantibody underwent an OGTT (time‐points −10, 0, 30, 60, 90 and 120 minutes) at their first study visit to assess the beta cell function before enrolment into the randomized trial. Participants positive for multiple autoantibodies (2 or more) underwent an IvGTT (time‐points −10, 0, 1, 3, 5, 7, 10, 30, 50, 70 and 90 minutes) at their first study visit. HbA1c was analysed in all participants. As the performance of the two tests for disposal of glucose is very different, since OGTT reflects the physiological uptake of glucose via the gut into the blood stream, while IvGTT reflects the disposal of glucose when injected intravenously, we only made a direct comparison from both tests for the fasting values.

K‐values were not calculated in Finnish participants due to the short IvGTT (−10, 0, 3, 5 and 10 minutes) performed in these participants.

### 2.3 Analysis of beta cell autoantibodies

GADA, IA‐2A and IAA were analysed using in‐house radio binding assays in two separate laboratories.^7^ Swedish samples were analysed at the CRC laboratory in Malmö and Finnish samples for ICA, IAA, IA‐2A and GADA were analysed in the DIPP Laboratory in Oulu and for ZnT8A in the PEDIA laboratory in Helsinki. Both GADA and IA‐2A assays in the Malmö Laboratory achieved high sensitivity and specificity in the last workshop in 2018. GADA achieved 64% sensitivity at specificity 94.4%, and IA‐2A achieved 62% sensitivity at specificity 100%. IAA achieved sensitivity 18% at specificity 96.7%. ZnT8(R)A achieved sensitivity 54% at specificity 100%, ZnT8(W)A achieved sensitivity 52% at specificity 100% and ZnT8(Q)A achieved sensitivity 40% at specificity 100%. In the last IASP workshop (2018), the DIPP Laboratory, GADA achieved 60% sensitivity at specificity 97.8%, IA‐2A achieved 76% sensitivity at specificity 100%, IAA achieved sensitivity 40% at specificity 96.7% and ICA achieved 84% sensitivity at specificity 87.8%.

ZnT8R/W/QA (three variants at position 325) were analysed in Malmö for Swedish participants ^7^ and in Helsinki for Finnish participants.

### 2.4 Analysis of glucose, insulin, C‐peptide and HbA1c levels

Plasma glucose, serum insulin and serum C‐peptide were all analysed using standard assays at local Departments of Clinical Chemistry at University Hospitals in Malmö, Oulu and Turku.

In Malmö, an enzymatic assay with absorbance measurement was used for measurement of p‐glucose (Roche Diagnostics, Basel, Switzerland). A one‐step immunometric sandwich assay, electrochemilumiscence immunoassay (ECLIA; Roche Diagnostics, Basel, Switzerland), was used for measurement of s‐insulin and s‐C‐peptide. In Malmö, HbA1c was analysed using a spectrophotometric assay (Capillary 3 Tera; Sebia, Paris, France).

In Oulu, plasma glucose was measured by an enzymatic hexokinase assay (ADVIA Chemistry XPT, Munich, Germany). Serum insulin and C‐peptide were analysed by two‐site sandwich immunoassay using chemiluminescent technology and ADVIA Centaur XPT equipment (Siemens, Munich, Germany). Blood HbA1c by a photometric method measuring HbA1c and the total haemoglobin and their ratio is reported (ADVIA Chemistry Hemoglobin A1c_3 reagents, Munich, Germany).

In Turku, an enzymatic assay with absorbance measurement for p‐glucose with Cobas c 702 (Roche Diagnostics, Basel, Switzerland) was used. An electrochemilumiscence immunoassay (ECLIA; Roche Diagnostics) was used for measurement of p‐insulin and p‐C‐peptide with a Cobas c 801, while a Cobas c 501 analyser (Roche Diagnostics GmbH) was used for HbA1c analysis.

The reference ranges for samples analysed were as follows:
Malmö: p‐glucose 1 month – 18 years: 3.3‐5.6 mmol/L, above 18 yrs: 4.2‐6.3 mmol/L, s‐insulin < 25 mIU/ L, s‐C‐peptide: 0.37‐1.5 nmol/ L.
Oulu: p‐glucose 4.2‐6.0 mmol/L, s‐insulin 5‐20 mU/L, s‐C‐peptide: 0.2‐1.0 nmol/ L
Turku: p‐glucose 4‐6 mmol/L, s‐insulin 2.6‐25 mU/L, s‐C‐peptide 0.37‐1.47 nmol/L.
All sample reference ranges apply to fasting condition.
The reference range for HbA1c analysed was as follows: Malmö 27‐42 mmol/mol,
Oulu 20‐42 mmol/mol and Turku 20‐42 mmol/mol.

### 2.5 Statistical methods

IBM SPSS statistics 25, was used for the statistical calculations. Student's t test was used to compare variables with normal distributions. Variables with non‐normal distributions or low number of participants were compared using Mann‐Whitney U test or Kruskal‐Wallis test, where appropriate. P‐values below 0.05 were considered as significant. Correlations were estimated using Pearson's correlation coefficient for normally distributed variables and Spearman's rank correlation coefficient for those at non‐normal distributions.

Areas under the curve (AUCs) for glucose, insulin and C‐peptide based on 5 time‐points in the OGTT (0, 30, 60, 90 and 120 minutes) were calculated using the auc function in the R‐package (MESS version 0.5.2 in R; www.r‐project.org) with linear interpolation using the trapezoidal rule. We only report AUCs based on complete data (5 measurements, 29 for glucose, 23 for insulin and 24 for C‐peptide). The analysis was stratified by sex, as well as by age, below and above 18 years, in order to examine the possible impact of these variables.

Fasting levels of glucose, insulin and C‐peptide were compared between participants with a single autoantibody and participants with multiple autoantibodies. Otherwise, we were not able to compare glucose, insulin and C‐peptide levels between participants positive for a single autoantibody and participants positive for multiple autoantibodies since those with single autoantibody underwent an OGTT at their first visit and those with multiple autoantibodies underwent an IvGTT.

## 3 RESULTS

### 3.1 The demographics of the study participants

A total of 76 participants positive for either a single or multiple autoantibodies underwent an OGTT or an IvGTT, 51 participants in Sweden and 25 participants in Finland. The islet autoantibody details of the participants are found in Supplementary online material (Supplementary online Table S1 and S2).

The participants were between 2‐49.99 years, but there were no participants between 18 and 30 years, and the median age of those under 18 years was 10 years.

The mean age, in both Sweden and in Finland, was older in participants who underwent an OGTT (mean 37.7 ± 8.72 years) and (mean 28.2 ± 16.0 years) as compared to those who underwent an IvGTT (mean 17.3 ± 11.8 years; *P* < .001) and (mean 8.87 ± 7.99 years; *P* < .001), respectively. Furthermore, participants from Finland who underwent an IvGTT were younger (mean 8.87 ± 7.99 years) than those from Sweden (mean 17.3 ± 11.8 years; *P* = .0140). There was no difference in age or BMI between men and women with multiple autoantibodies in Sweden nor in Finland (Table 1).

**Table 1:** Clinical characteristics of participants enrolled in the TEFA study in Sweden and in Finland

Variable	Sweden				Finland			
	n	Value (mean ± SD)	*P*	n		Value (mean ± SD)	*P*
Single AAB	21	N/A	N/A	9		N/A	N/A
Age (y)	21	37.7 ± 8.72	N/A	9		28.2 ± 16.0	N/A
Women	10	37.8 ± 8.78	0.778	8		30.8 ± 15.0	N/A
Men	11	37.6 ± 9.10		1		7.41 ± 0.00	
Multiple AAB	30	N/A	N/A	16		N/A	N/A
Age (y)	30	17.3 ± 11.8	N/A	16		8.87 ± 7.99	N/A
Women	16	20.1 ± 12.9	0.167	8		12.31 ± 9.93	0.084
Men	14	14.1 ± 9.96		8		5.42 ± 3.34	
BMI (kg/m^2^)	30	21.3 ± 4.59	N/A	15		16.6 ± 2.72	N/A
Women	16	22.4 ± 4.88	0.175	8		17.0 ± 3.46	0.628
Men	14	20.1 ± 4.06		7		16.2 ± 1.74	

Note

Participants with a single autoantibody (AAB) (n = 30) performed an oral glucose tolerance test (OGTT), participants with multiple autoantibodies (n = 46) performed an Intravenous glucose tolerance test (IvGTT).

In Swedish participants positive for a single autoantibody, HbA1c values were higher in men (34.7 ± 2.71 mmol/mol) than in women (31.8 ± 2.25 mmol/mol; *P* = .0180). There were too few participants in Finland to perform this comparison. There was no difference in HbA1c, vitamin D levels or glucose between men and women in Sweden or in Finland or when participant below and above 18 years were compared. (Supplementary online Table S3).

### 3.2 OGTT in single autoantibody participants

As of 15 February 2019, a total of 30 participants positive for a single autoantibody had undergone an OGTT at their first visit, 21 in Sweden and 9 in Finland. One participant in Finland presented with two islet autoantibodies at the screening visit and was diagnosed with type 1 diabetes at the visit based on OGTT. The remaining 29 participants with only one autoantibody had normal glucose tolerance with the highest reported 2h‐value for glucose of 7.70 mmol/L. Insulin responses to the glucose challenge on OGTT ranged between 13.0 and 143 mIU/L after 120 minutes with C‐peptide values between 0.74 and 4.60 nmol/L. Additional details on glucose, insulin and C‐peptide values from OGTT can be found in Supplementary online Table S4a‐c and Supplementary online Figures S1‐S3.

There was no difference in AUC for glucose from OGTT in Swedish and Finnish participants (*P* = .518; Table 2). AUC for insulin from OGTT was higher in the Finnish participants (n = 6; 11 676 ± 8356) than in the Swedish participants (n = 17; 5333 ± 3194; *P* = .0360; Table 2). AUC for C‐peptide was similar in Swedish and Finnish participants (*P* = .836; Table 2). There were no differences in AUC of glucose, insulin or C‐peptide with respect to gender. No differences were identified in AUC of glucose, insulin or C‐peptide in participants below or above 18 years of age in Sweden (Supplementary online Table S5). There were no differences in glucose values at any time‐point in participants positive for GADA alone (n = 10) or IAA alone (n = 11) in Sweden (Supplementary online Table S6). This analysis was not performed separately for participants from Finland due to too few participants in each group. There was only one participant from Finland that was positive for IA‐2A alone and none in Sweden.

**Table 2:** HbA1c and area under the curve (AUC) from oral glucose tolerance test (OGTT) from participants enrolled in the TEFA study with a single autoantibody (AAB) (n = 30) in both Sweden and Finland

Variable	Glucose			Insulin			C‐Peptide		
	n (%)	Value (mean ± SD)	*P*	n (%)	Value (mean ± SD)	*P*	n (%)	Value (mean ± SD)	*P*
HbA1C (mmol/ml)									
Sweden	20 (67%)	34.1 ± 3.44							
Finland	9 (30%)	34.8 ± 6.12							
AUC									
Sweden	21 (70%)	725 ± 132	.581	17 (57%)	5332 ± 3194	.0360	19 (63%)	422 ± 78.2	.836
Finland	8 (27%)	753 ± 125		6 (20%)	11 675 ± 8356		5 (17%)	212 ± 69.7	

Abbreviation: AUC, Area under the curve.

No differences in glucose metabolism were detected among those with a single autoantibody, either GADA (n = 17) or IAA (n = 13), with respect to autoantibody levels. The tested parameters were HbA1c, −10 minutes glucose, −10 minutes C‐peptide and 120 minutes insulin, 120 minutes glucose, 120 minutes C‐peptide and 120 minutes insulin (Supplementary online Table S7).

### 3.3 IvGTT in participants with multiple autoantibodies

A total of 46 participants positive for multiple autoantibodies underwent an IvGTT at their first visit, 30 in Sweden and 16 in Finland. Fasting glucose varied from 4.00 mmol/L to 7.40 mmol/L in Swedish participants and from 3.50 mmol/L to 6.40 mmol/L in Finnish participants.

Further details of the glucose, insulin and C‐peptide levels can be found in Supplementary online Table S8a, b and c and Supplementary online Figure S4a, b, S5a and b and S6a and b.

There was no difference in K‐values between men and women in Sweden, neither in children below and above 10 years in Sweden or when participants below and above 18 years were compared (Supplementary online Table S3). K‐values were not calculated in Finnish participants due to the short IvGTT performed in these participants. There was no difference in glucose levels at any time‐point in participants below and above 18 years of age (Supplementary online Table S9).

There was no difference in glucose values at −10 minutes or FPIR between men and women in Sweden or in Finland, neither in FPIR in children below and above 10 years in Sweden or in Finland or when participant below and above 18 years were compared. (Supplementary online Table S3).

FPIR was lower in participants with three or more autoantibodies (n = 13) compared to those with only two autoantibodies (n = 15) in Sweden (*P* = .0330) but not in Finland (n = 5). K‐values were similar between those with two autoantibodies as compared to those with three or more (Table 3).

**Table 3:** First‐phase insulin response (FPIR), K‐value and white blood cells in relation to number of autoantibodies in participants enrolled in the TEFA study with multiple autoantibodies (AAB) (n = 46) both in Sweden and in Finland (2 AAB = 2, 3 AAB = 3 or more autoantibodies). (K‐value was not obtained in Finland due to the short IvGTT)

Variable	Sweden			Finland		
	n (%)	Median; range	*P*	n (%)	Median; range	*P*	
FPIR							
2 AAB	15 (33%)	146 (19.0 ‐ 545)	.0330	5 (11%)	46.8 (13.2 ‐ 93.3)	.841	
3 AAB	13 (28%)	83.0 (20.0 ‐ 343)		5 (11%)	62.5 (12.0 ‐ 74.5)		
K‐value							
2 AAB	16 (33%)	2.08 (1.20 ‐ 3.47)	.131				
3 AAB	14 (30%)	1.72 (0.85 ‐ 4.13)					

At time‐point −10 minutes in the Swedish participants, there were significant correlations between BMI and fasting insulin levels (*P* = .0170) and BMI and fasting C‐peptide levels (*P* = .0160), but not between BMI and fasting glucose levels. In the Finnish participants, there were significant correlations between BMI and fasting glucose levels (*P* = .0410) and BMI and fasting C‐peptide levels (*P* = .0180), but no significant correlation between BMI and fasting insulin levels (Table 4).

**Table 4:** Correlations between body mass index (BMI), glucose, insulin and C‐peptide and first‐phase insulin response (FPIR), K‐value from intravenous glucose tolerance test (IvGTT) of participants enrolled in the TEFA study with multiple autoantibodies (AAB) (n = 46) both in Sweden and in Finland. (BMI in relation to K‐value was not obtained in Finland due to the short IvGTT)

Variable	Sweden			Finland		
	n (%)	Correlation	*P*	n (%)	Correlation	*P*
BMI – glucose (mmol/L) M10	30 (65%)	*r* = 0.0490	.769	15 (33%)	r = 0.532	.0410
BMI – Insulin (mlU/L) M10	30 (65%)	*r* = 0.439	.0170	8 (17%)	r = −0.161	.704
BMI – C‐Peptide (nmol/L) M10	30 (65%)	*r* = 0.445	.0160	8 (17%)	r = 0.796	.0180
BMI – FPIR	28 (67%)	*r* = 0.114	.562	9 (20%)	r = −0.302	.429
BMI – K‐value	30 (61%)	*r* = −0.224	.235			

Abbreviation: M10 = 10 minutes prior to ingestion of glucose.

### 3.4 Comparison of fasting levels of glucose, insulin and C‐peptide between participants with a single autoantibody or with multiple autoantibodies

There was no difference in fasting glucose levels between participants with two or three and more autoantibodies, neither in Swedish nor in Finnish participants. Fasting insulin levels were higher in participants with multiple autoantibodies (median 12.0 mIU/L; range 3.00‐28.0 mlE/L) as compared with those positive only for a single autoantibody (median 7.50 mIU/L; range 2.00‐20.0 mlE/L; *P* = .0300) in the Swedish participants, but there was no difference between these two groups in the Finnish participants. In contrast, fasting C‐peptide levels were significantly higher in participants with a single autoantibody (median 0.45 nmol/L; range 0.22‐0.56) as compared to those with multiple autoantibodies (median 0.21 nmol/L; range 0.11‐0.38; *P* = .0180) in the Finnish participants, but there was no difference in the Swedish participants (Table 5).

**Table 5:** Metabolic results of participants enrolled in the TEFA study with a single autoantibody (AAB) (n = 30) and with multiple autoantibodies (n = 46) both in Sweden and in Finland

Variable	Sweden			Finland		
	n (%)	Median; range	*P*	n (%)	Median; range	*P*
Glucose (mmol/L) M10						
Single AAB	21 (70%)	5.30 (4.60 ‐ 6.20)	.185	9 (30%)	5.30 (4.30 ‐ 6.80)	.207
Multiple AAB	30 (65%)	5.40 (4.00 ‐ 7.40)		16 (35%)	5.05 (3.50 ‐ 6.40)	
Insulin (mIU/L) M10						
Single AAB	18 (60%)	7.50 (2.00 ‐ 20.0)	.0300	7 (23%)	11.4 (2.20 ‐ 16.8)	.252
Multiple AAB	29 (63%)	12.0 (3.00 ‐ 28.0)		9 (20%)	5.10 (1.20 ‐ 21.9)	
C‐Peptide (nmol/L) M10						
Single AAB	18 (60%)	0.69 (0.43 ‐ 1.40)	.948	6 (20%)	0.45 (0.22 ‐ 0.56)	.0180
Multiple AAB	29 (63%)	0.67 (0.38 ‐ 1.30)		9 (20%)	0.21 (0.11 ‐ 0.38)	

Abbreviation: M10 = 10 minutes prior to ingestion of glucose.

## 4 DISCUSSION

The most important finding of this study was that all participants with only a single autoantibody had normal beta cell function based on glucose, C‐peptide and insulin levels in the 6‐point OGTT test. Therefore, none of the participants with a single autoantibody was eligible to enter the randomized trial with either gluten free or regular diet. This finding is in line with a recent study reporting that single islet autoantibody at clinical diagnosis of type 1 diabetes is associated with older age in the patients as well as insulin resistance and higher BMI.^8^ It was important to test single autoantibody‐positive participants as 15% (483/3165) newly diagnosed 1‐ to 18‐year‐olds had only one islet autoantibody at the time of clinical diagnosis.^9^ Therefore, it could not be excluded that single autoantibody‐positive participants with impaired glucose tolerance would have been eligible for the TEFA study.

Interestingly, HbA1c levels were higher but still within the reference range in men with a single autoantibody as compared with women in Swedish participants, but no difference in age was observed. In some studies, HbA1c has been proven to be a useful marker to predict the progression to type 1 diabetes.^4^, ^10^ An increase in HbA1c levels has previously been reported as a specific but not sensitive indicator of progression to type 1 diabetes in children.^10^ In a study of children with multiple autoantibodies, it was shown that a 10% increase in HbA1c was highly predictive for type 1 diabetes within one year.^4^ Fasting insulin levels were higher in participants with multiple autoantibodies as compared to those with a single autoantibody in the Swedish participants. This finding is in contrasts to what has been reported from the TEDDY study where reduced fasting insulin levels in children with multiple autoantibodies were predictive for progression to type 1 diabetes in children aged 3 to 6 years of age.^11^ In contrast, fasting C‐peptide levels were significantly higher in participants with a single autoantibody as compared to those with multiple autoantibodies in the Finnish participants but there was no difference in the Swedish participants. These participants might have been in different phases of progression to type 1 diabetes; the Finnish participants with multiple autoantibodies may have been closer to clinical diagnosis than the Swedish participants. An additional explanation to this finding might be the low number of participants in Finland and the difference in the age groups between Sweden and Finland. In Finland, the median age was lower than in Sweden, while the age range was wider in Sweden than in Finland.

BMI was significantly correlated to fasting C‐peptide in both the Swedish and the Finnish participants, while BMI was significantly correlated to fasting insulin in the Swedish participants only and to fasting glucose in the Finnish participants. It has previously been shown that there is a correlation between BMI and C‐peptide in healthy children ^12^ as well as in patients newly diagnosed with type 1 diabetes, both in paediatric cases ^13^ and in adult cases.^14^ The correlation between BMI and insulin in the Swedish participants, but BMI and glucose in the Finnish participants, is difficult to explain. Possibly, the difference in the age groups between Sweden and Finland, and the low number of participants in Finland, might have had an impact on these outcomes.

Another important finding was that participants who were positive for three or more autoantibodies had significantly lower FPIR as compared to participants positive for only two autoantibodies. This finding indicates that the higher the number of autoantibodies, the more severe is the disturbance in insulin production. A higher number of autoantibodies is an indication of an approaching manifest type 1 diabetes, a finding supported in other studies.^15^, ^16^ Declined FPIR was found several years before the onset of clinical type 1 diabetes in the DIPP children with multiple autoantibodies but not in those with a single autoantibody.^17^, ^18^, ^19^

The strength of the present study is that all participants (except one) were persistently islet autoantibody positive prior to enrolment into this study and that all participants followed a coordinated protocol. A major limitation on the present study is that participants, positive for a single autoantibody underwent an OGTT, while participants, positive for multiple autoantibodies underwent an IvGTT. Thus, the outcome of these glucose challengers is not comparable. Another limitation of the present study is that measurements of glucose, insulin and C‐peptide were analysed in three different clinical laboratories with different reference values. Differences in assay performances therefore precluded direct comparisons between the three different laboratories. An additional limitation is that autoantibody measurement, both prior to and during the study, were performed in two different laboratories (one for each country). Another major limitation is the low number of participants raising concerns for statistical power; nevertheless, it requires a large screening population of healthy participants to identify autoantibody‐positive participants. In Sweden, a total of 3738 family members were screened, and in Finland, a total of 1526 family members were screened, to a participant enrolled in the TEDDY study.

In conclusion, participants with only a single autoantibody appeared to have a normal beta cell function. Participants positive for three or more autoantibodies have a lower FPIR as compared to participants positive for two autoantibodies, indicating a more severe beta cell lesion.

## CONFLICT OF INTEREST

The authors declare that there is no conflict of interests associated with this study.

## AUTHOR CONTRIBUTIONS

ÅL and HEL conceived the original idea. MMM and CT researched the data and drafted the manuscript. MMM and FS contributed to study participant recruitment. CT performed conventional statistical analyses using SPSS, MM performed parts of the statistical analyses using the R‐project. FS, ÅL, HEL, JK, JJK, JT, RV, PT, ML, HB, AK reviewed/edited the manuscript. All authors approved the final version of the manuscript for submission.

## Supporting information

Supplementary Material

Supplementary Material

## Data Availability

The data that support the findings of this study are available on request from the corresponding author. The data are not publicly available due to privacy or ethical restrictions.

## References

[1] Siljander HT , Hermann R , Hekkala A , et al. Insulin secretion and sensitivity in the prediction of type 1 diabetes in children with advanced beta‐cell autoimmunity. Eur J Endocrinol. 2013;169(4):479‐485. 23904276 10.1530/EJE-13-0206

[2] Elding Larsson H , Larsson C , Lernmark A , DiAPREV‐IT study group . Baseline heterogeneity in glucose metabolism marks the risk for type 1 diabetes and complicates secondary prevention. Acta Diabetol. 2015;52(3):473‐481. 25381193 10.1007/s00592-014-0680-1

[3] Helminen O , Aspholm S , Pokka T , et al. OGTT and random plasma glucose in the prediction of type 1 diabetes and time to diagnosis. Diabetologia. 2015;58(8):1787‐1796. 25985749 10.1007/s00125-015-3621-9

[4] Helminen O , Aspholm S , Pokka T , et al. HbA1c Predicts Time to Diagnosis of Type 1 Diabetes in Children at Risk. Diabetes. 2015;64(5):1719‐1727. 25524912 10.2337/db14-0497

[5] Bingley PJ , Boulware DC , Krischer JP . The implications of autoantibodies to a single islet antigen in relatives with normal glucose tolerance: development of other autoantibodies and progression to type 1 diabetes. Diabetologia. 2016;59(3):542‐549. 26676824 10.1007/s00125-015-3830-2 PMC4742489

[6] Ziegler AG , Rewers M , Simell O , et al. Seroconversion to multiple islet autoantibodies and risk of progression to diabetes in children. JAMA. 2013;309(23):2473‐2479. 23780460 10.1001/jama.2013.6285 PMC4878912

[7] Delli AJ , Vaziri‐Sani F , Lindblad B , et al. Zinc transporter 8 autoantibodies and their association with SLC30A8 and HLA‐DQ genes differ between immigrant and Swedish patients with newly diagnosed type 1 diabetes in the Better Diabetes Diagnosis study. Diabetes. 2012;61(10):2556‐2564. 22787139 10.2337/db11-1659 PMC3447907

[8] Redondo MJ , Sosenko J , Libman I , et al. Single Islet Autoantibody at Diagnosis of Clinical Type 1 Diabetes is Associated with Older Age and Insulin Resistance. J Clin Endocrinol Metab. 2019;105(5):1629‐1640. 10.1210/clinem/dgz296 PMC7089846 31867614

[9] Andersson C , Kolmodin M , Ivarsson SA , et al. Islet cell antibodies (ICA) identify autoimmunity in children with new onset diabetes mellitius negative for other islet cell antibodies. Pediatr Diabetes. 2014;15(5):336‐44. 24206368 10.1111/pedi.12093

[10] Vehik K , Cuthbertson D , Boulware D , et al. Performance of HbA1c as an early diagnostic indicator of type 1 diabetes in children and youth. Diabetes Care. 2012;35(9):1821‐1825. 22699293 10.2337/dc12-0111 PMC3425003

[11] Jacobsen LM , Larsson HE , Tamura RN , et al. Predicting progression to type 1 diabetes from ages 3 to 6 in islet autoantibody positive TEDDY children. Pediatr Diabetes. 2019;20(3):263‐270. 30628751 10.1111/pedi.12812 PMC6456374

[12] Huus K , Akerman L , Raustorp A , Ludvigsson J . Physical Activity, Blood Glucose and C‐Peptide in Healthy School‐Children, a Longitudinal Study. PLoS One. 2016;11(6):e0156401. 27270732 10.1371/journal.pone.0156401 PMC4896630

[13] Sabek OM , Redondo MJ , Nguyen DT , et al. Serum C‐peptide and osteocalcin levels in children with recently diagnosed diabetes. Endocrinol Diabetes Metab. 2019;3(1):e00104. 31922031 10.1002/edm2.104 PMC6947692

[14] Törn C , Landin‐Olsson M , Lernmark Å , et al. Prognostic factors for the course of B‐cell function in autoimmune diabetes. J Clin Endocrinol Metab. 2000;85:4619‐4623. 11134117 10.1210/jcem.85.12.7065

[15] Ling Q , Lu J , Li J , Xu Q , Zhu D , Bi Y . Risk of beta‐cell autoimmunity presence for progression to type 1 diabetes: A systematic review and meta‐analysis. J Autoimmun. 2018;86:9‐18. 28988642 10.1016/j.jaut.2017.09.012

[16] Jacobsen LM , Bocchino L , Evans‐Molina C , et al. The risk of progression to type 1 diabetes is highly variable in individuals with multiple autoantibodies following screening. Diabetologia. 2019;63(3):588‐596. 31768570 10.1007/s00125-019-05047-w PMC7229995

[17] Koskinen MK , Helminen O , Matomaki J , et al. Reduced beta‐cell function in early preclinical type 1 diabetes. Eur J Endocrinol. 2016;174(3):251‐259. 26620391 10.1530/EJE-15-0674 PMC4712442

[18] Koskinen MK , Lempainen J , Loyttyniemi E , et al. Class II HLA Genotype Association With First‐Phase Insulin Response Is Explained by Islet Autoantibodies. J Clin Endocrinol Metab. 2018;103(8):2870‐2878. 29300921 10.1210/jc.2017-02040 PMC6097602

[19] Koskinen MK , Mikk ML , Laine AP , et al. Longitudinal Pattern of First‐Phase Insulin Response Is Associated With Genetic Variants Outside the Class II HLA Region in Children With Multiple Autoantibodies. Diabetes. 2020;69(1):12‐19. 31591105 10.2337/db19-0329

