# Supplementary material for: Beta cell function in participants with single or multiple islet autoantibodies at baseline in the TEDDY Family Prevention Study: TEFA
Source: Endocrinol Diabetes Metab. 2020 Nov 5;4(2):e00198. doi: 10.1002/edm2.198 (PMC8029501; doi:10.1002/edm2.198)
Supplement: Supplementary file 1 — Supplementary Material [file EDM2-4-e00198-s002.pdf]

Figure 1 - 3. Oral glucose tolerance test (OGTT) glucose, insulin and C-peptide results for subjects enrolled in the TEFA-study with a single autoantibody (n=30) in Sweden and Finland. Reference values in fasting condition for glucose: ≤6,1 mmol/L and at 120 min ≤7,8 mmol/L, for insulin: min <25mlE/L in Sweden and 2.6-25 mU/L in Finland and for C-peptide: 0,37-1.5 nmol/L in Sweden and in Turku and >0.9 nmol/L in Oulu.

Figure 1.

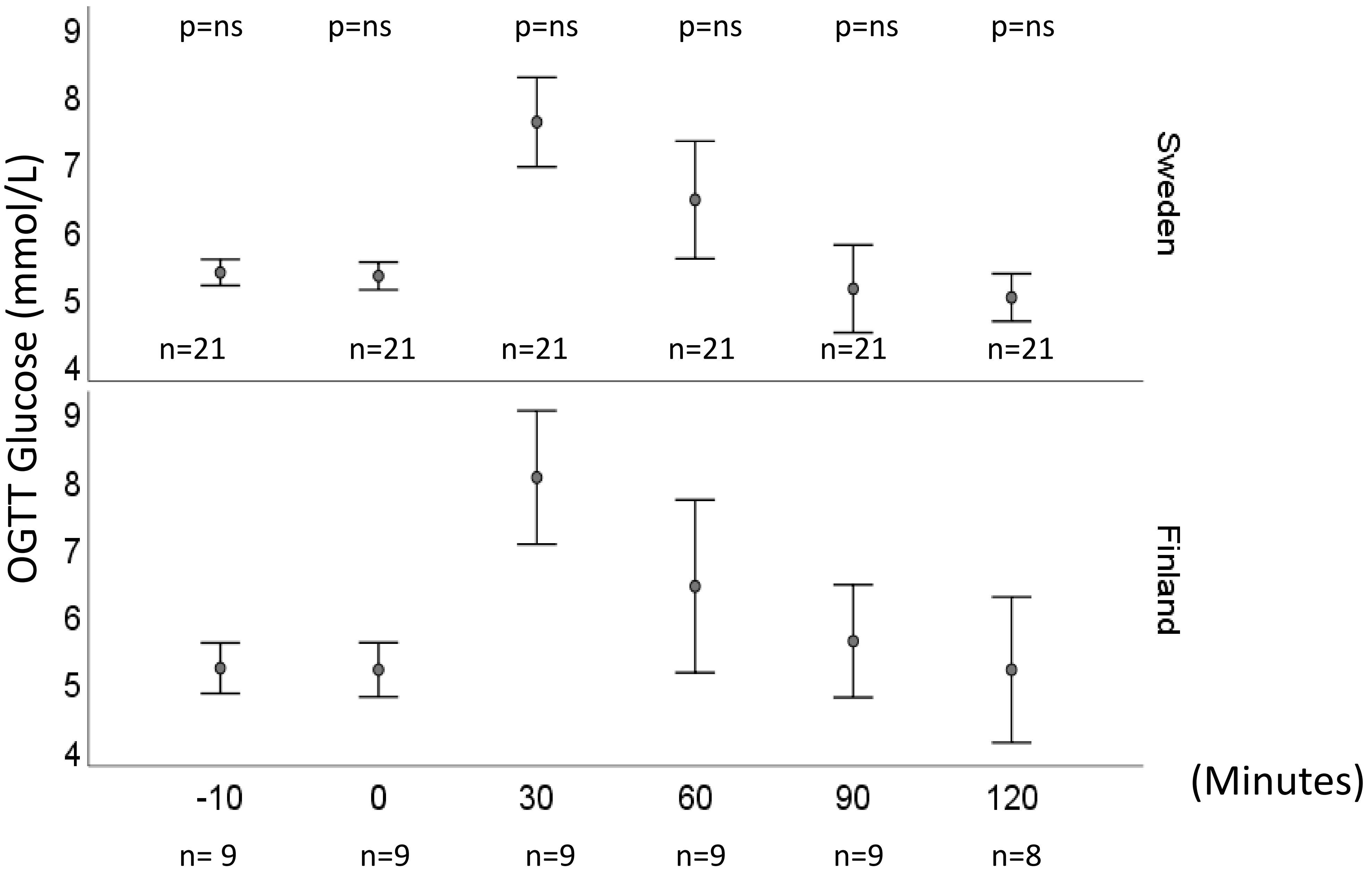

p=ns
p=ns
p=ns
p=ns
p=ns
p=ns
n=21
n=21
n=21
n=21
n=21
n=21
Sweden
Finland
OGTT Glucose (mmol/L)
9
8
7
6
5
4
-10
0
30
60
90
120
(Minutes)
n= 9
n=9
n=9
n=9
n=9
n=8

Figure 2.

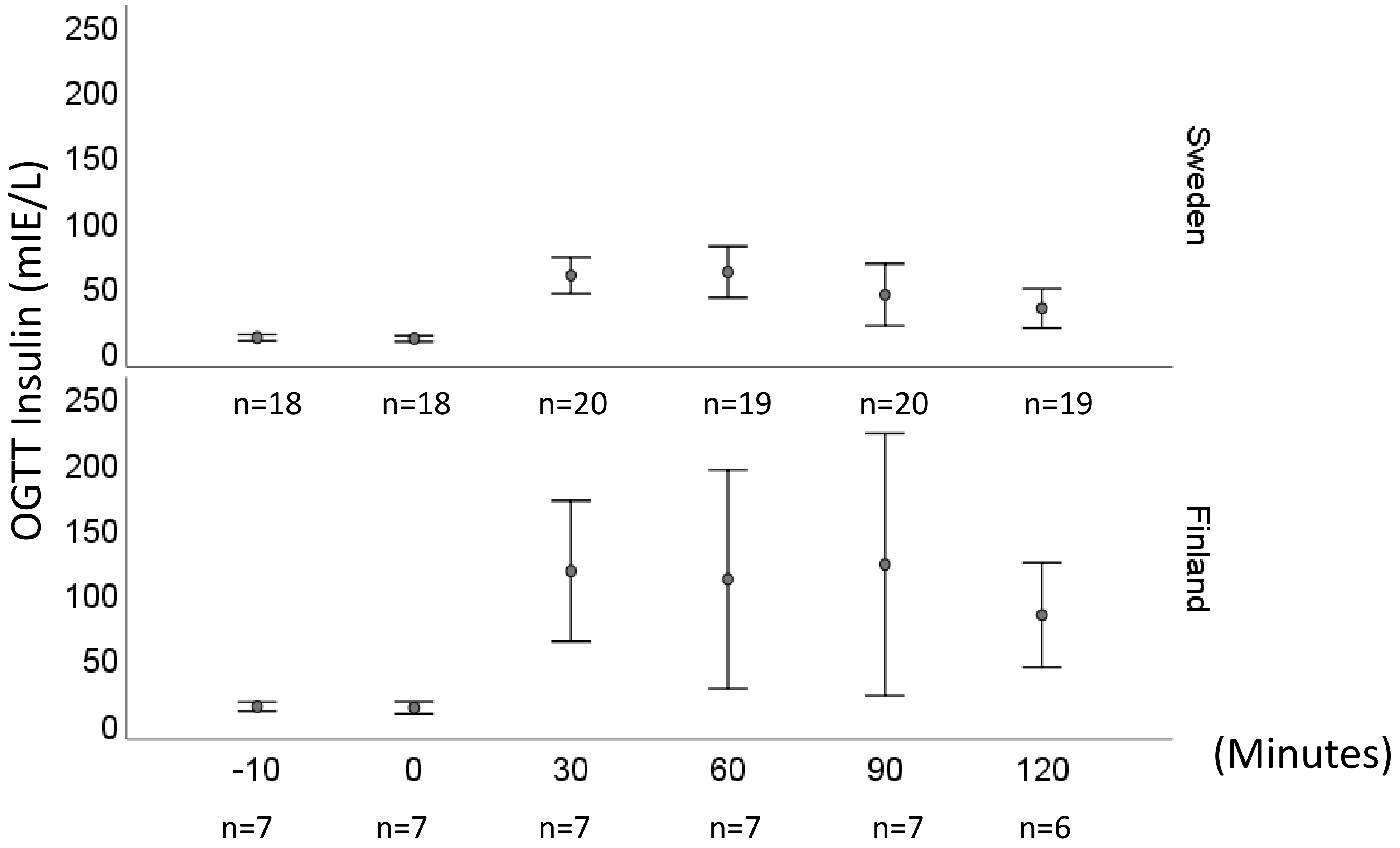
OGTT Insulin (mIE/L)
250
200
150
100
50
0
Sweden
n=18
n=18
n=20
n=19
n=20
n=19
250
200
150
100
50
0
Finland
-10
0
30
60
90
120
(Minutes)
n=7
n=7
n=7
n=7
n=7
n=6

Figure 3.

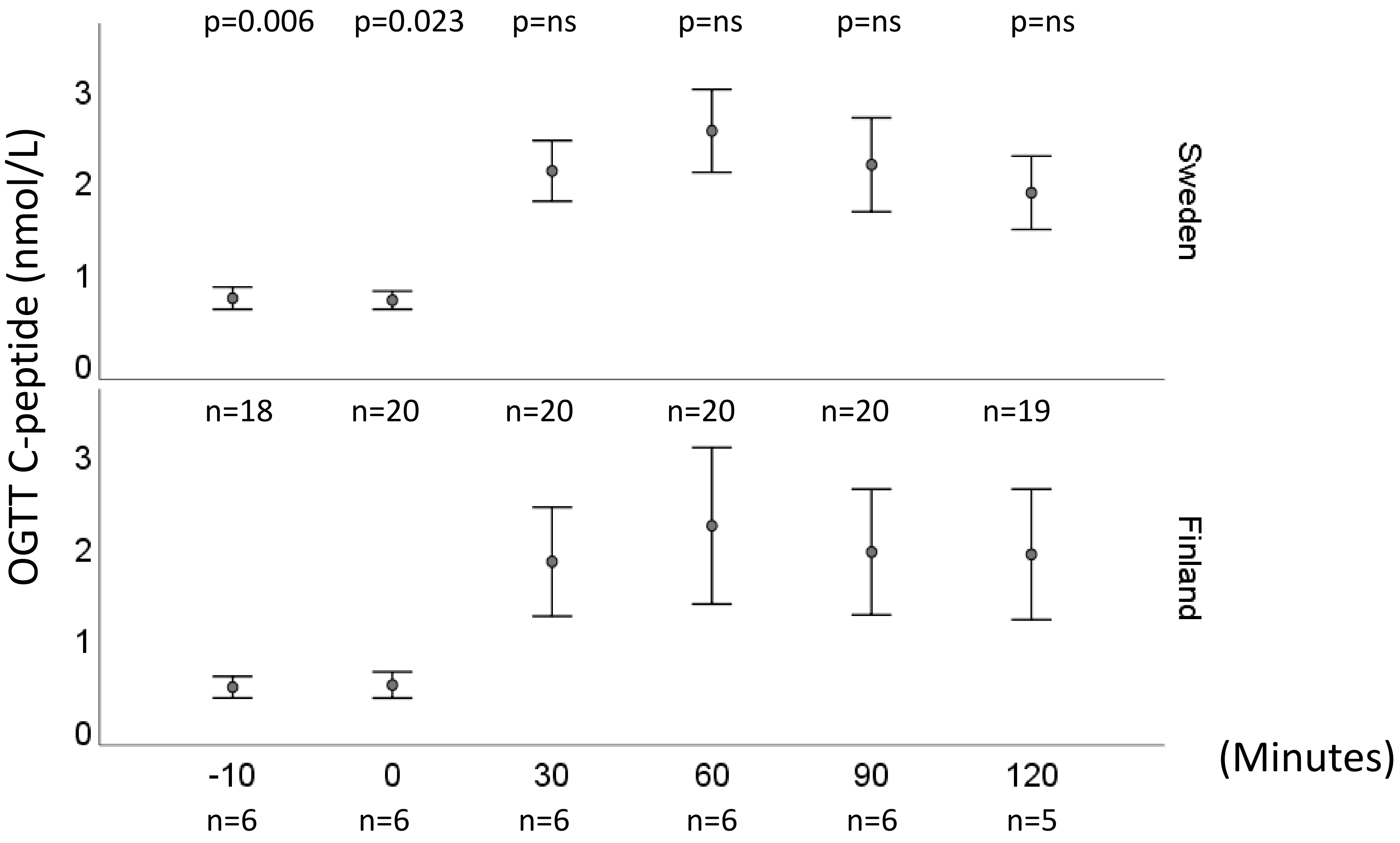

p=0.006
p=0.023
p=ns
p=ns
p=ns
p=ns
OGTT C-peptide (nmol/L)
0
1
2
3
Sweden
n=18
n=20
n=20
n=20
n=20
n=19
Finland
-10
0
30
60
90
120
(Minutes)
n=6
n=6
n=6
n=6
n=6
n=5

Figure 4 A, B - 6 A, B. Intravenous glucose tolerance test (IvGTT) glucose, insulin and C-peptide results for subjects enrolled in the TEFA-study with multiple autoantibodies (n=46) in Sweden and Finland. Reference values in fasting condition for glucose: ≤6,1 mmol/L and 90 min ≤7,8 mmol/L, for insulin: min <25mlE/L in Sweden and 2.6-25 mU/L in Finland and for C-peptide: min 0.37-1.5 nmol/L in Sweden and Turku and >0.9 nmol/L in Oulu. (Due to the short IvGTT the minutes 7, 30, 50, 70 and 90 were not obtained in Finland).

Figure 4A.

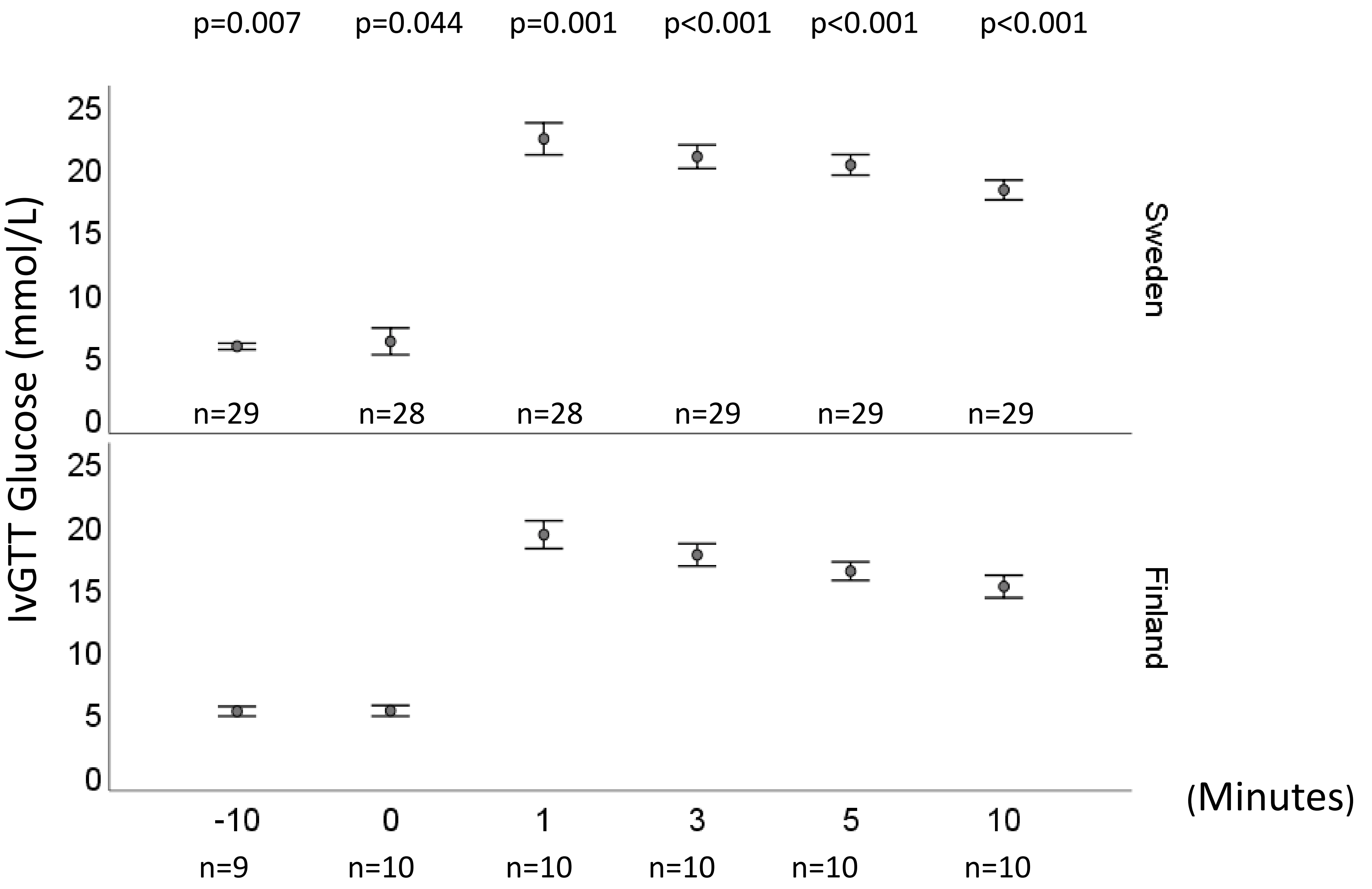
p=0.007
p=0.044
p=0.001
p<0.001
p<0.001
p<0.001
IvGTT Glucose (mmol/L)
25
20
15
10
5
0
Sweden
Finland
n=29
n=28
n=28
n=29
n=29
n=29
-10
0
1
3
5
10
(Minutes)
n=9
n=10
n=10
n=10
n=10
n=10

Figure 4B.

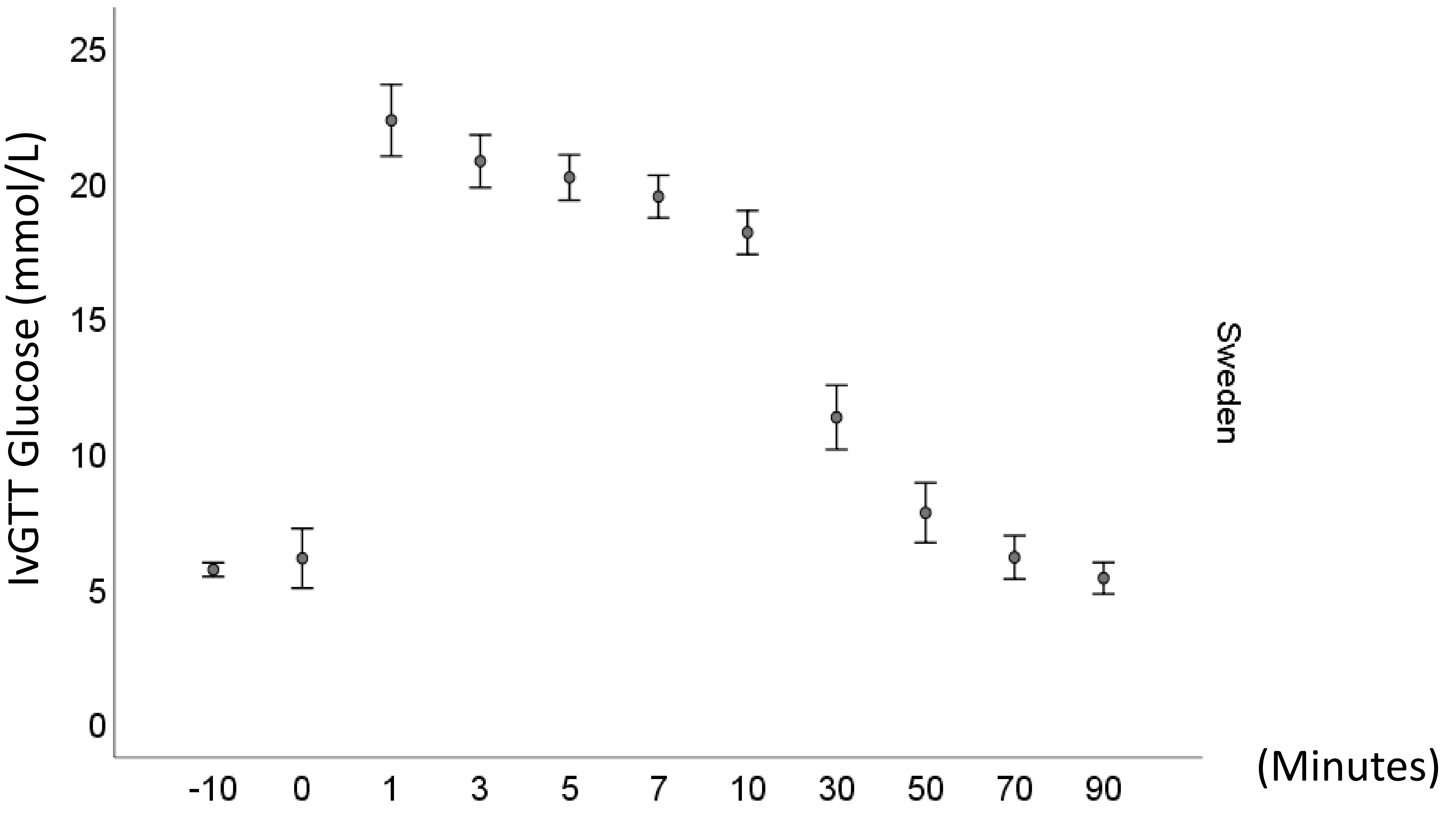
IvGTT Glucose (mmol/L)
25
20
15
10
5
0
-10
0
1
3
5
7
10
30
50
70
90
(Minutes)
Sweden

Figure 5A.

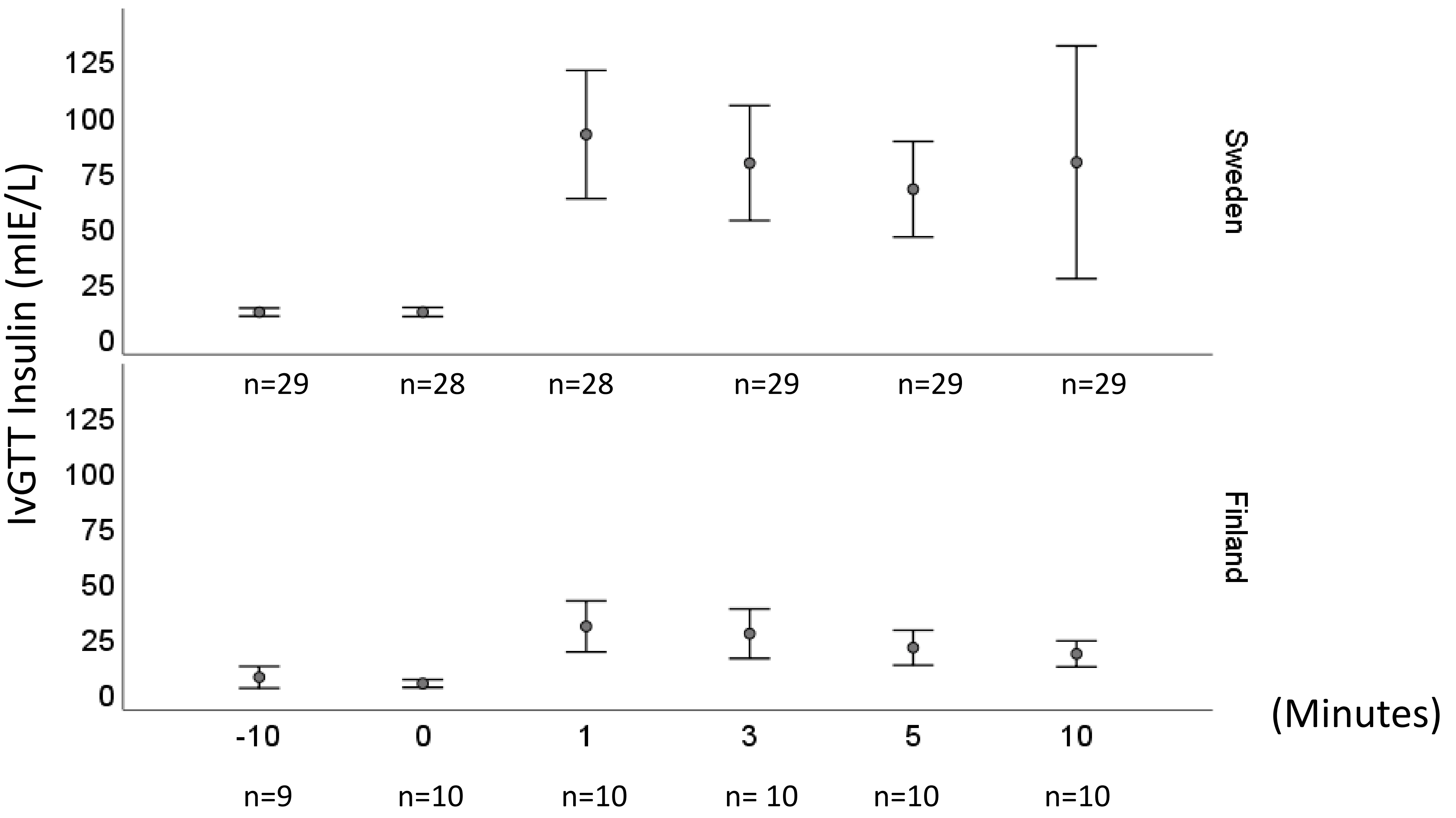

IvGTT Insulin (mIE/L)
125
100
75
50
25
0
Sweden
n=29
n=28
n=28
n=29
n=29
n=29
125
100
75
50
25
0
Finland
-10
0
1
3
5
10
(Minutes)
n=9
n=10
n=10
n= 10
n=10
n=10

Figure 5B.

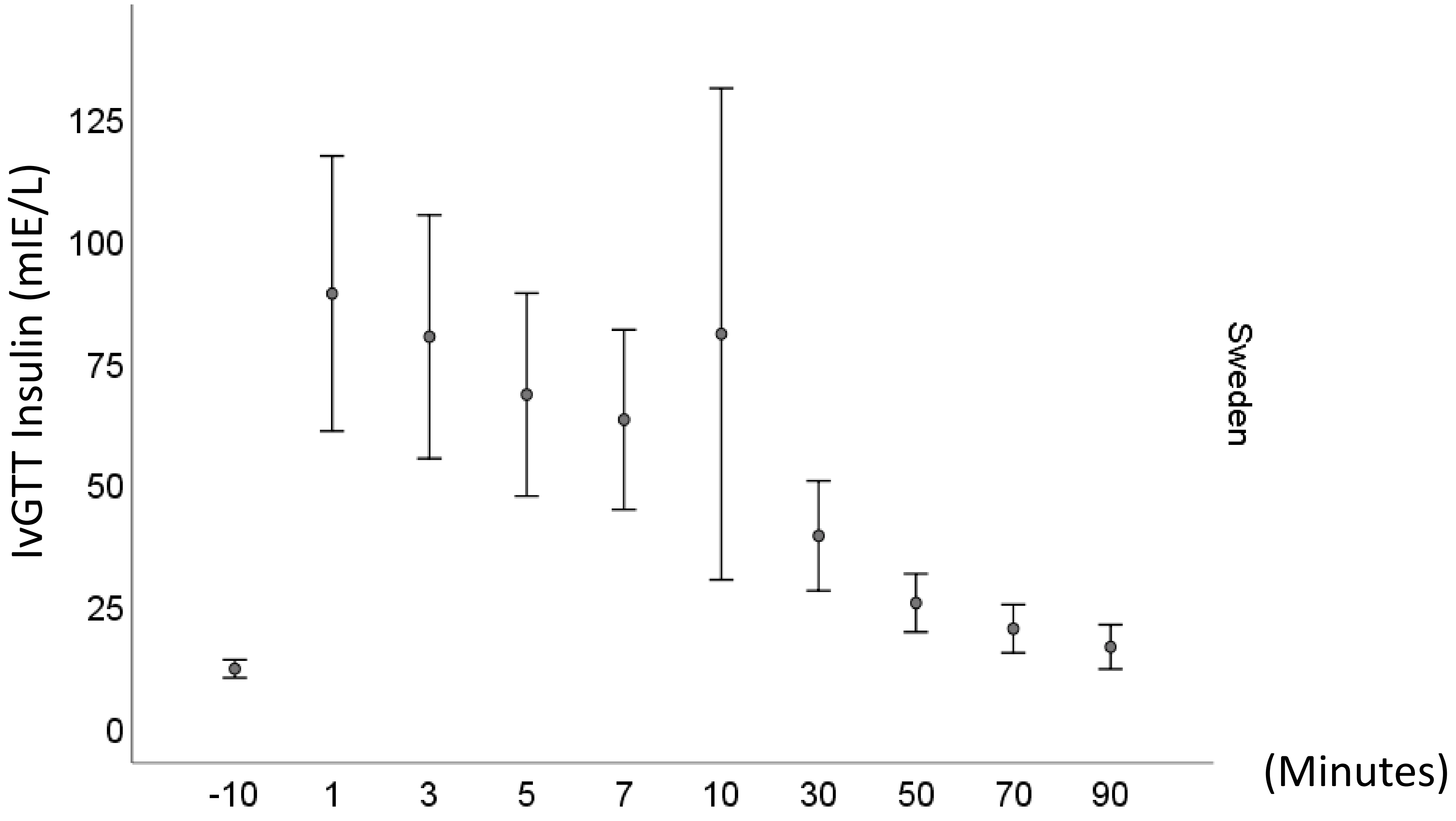
IvGTT Insulin (mIE/L)
125
100
75
50
25
0
-10
1
3
5
7
10
30
50
70
90
(Minutes)
Sweden

Figure 6A.

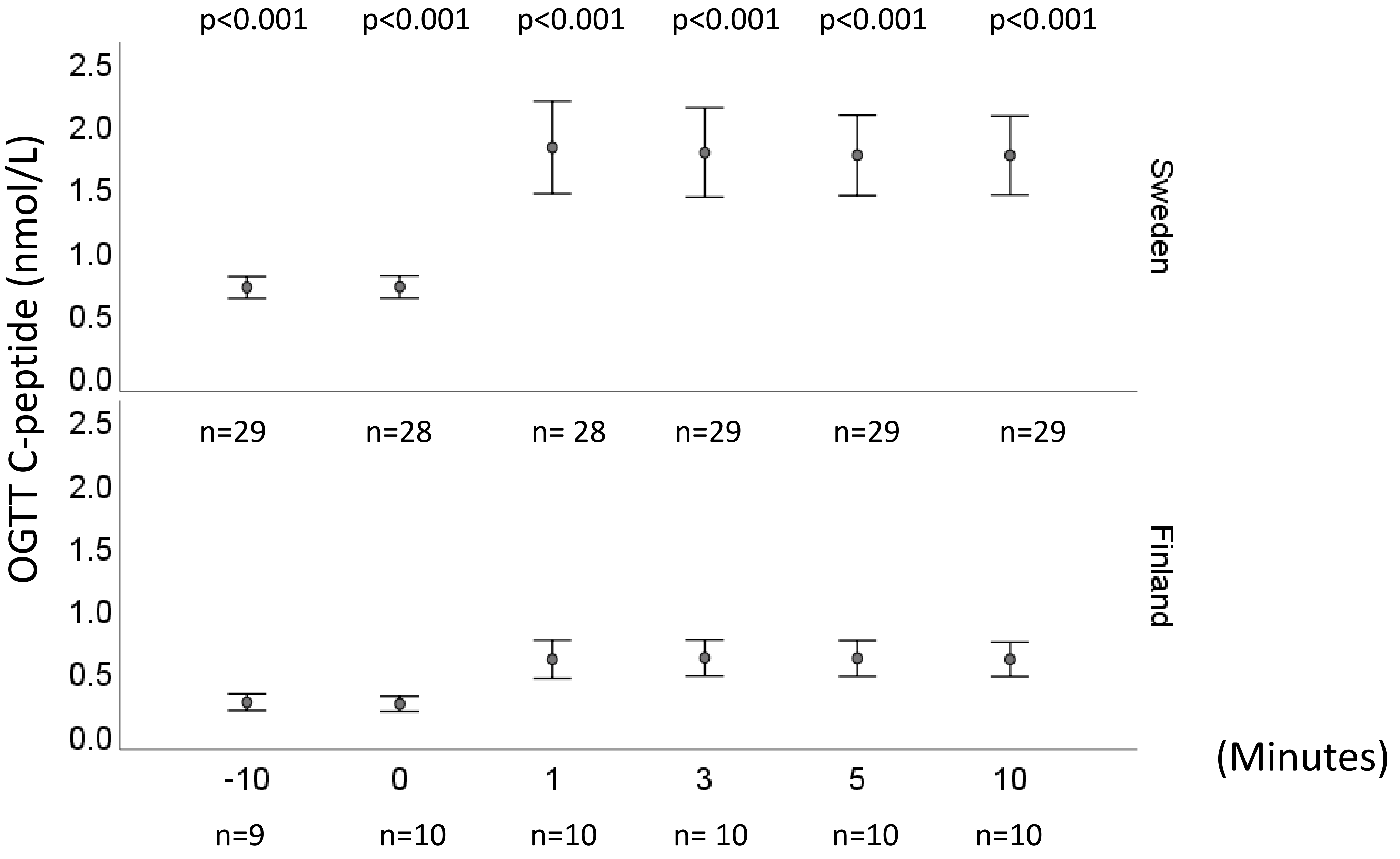
p<0.001
p<0.001
p<0.001
p<0.001
p<0.001
p<0.001
OGTT C-peptide (nmol/L)
2.5
2.0
1.5
1.0
0.5
0.0
Sweden
n=29
n=28
n= 28
n=29
n=29
n=29
Finland
(Minutes)
-10
0
1
3
5
10
n=9
n=10
n=10
n= 10
n=10
n=10

Figure 6B.

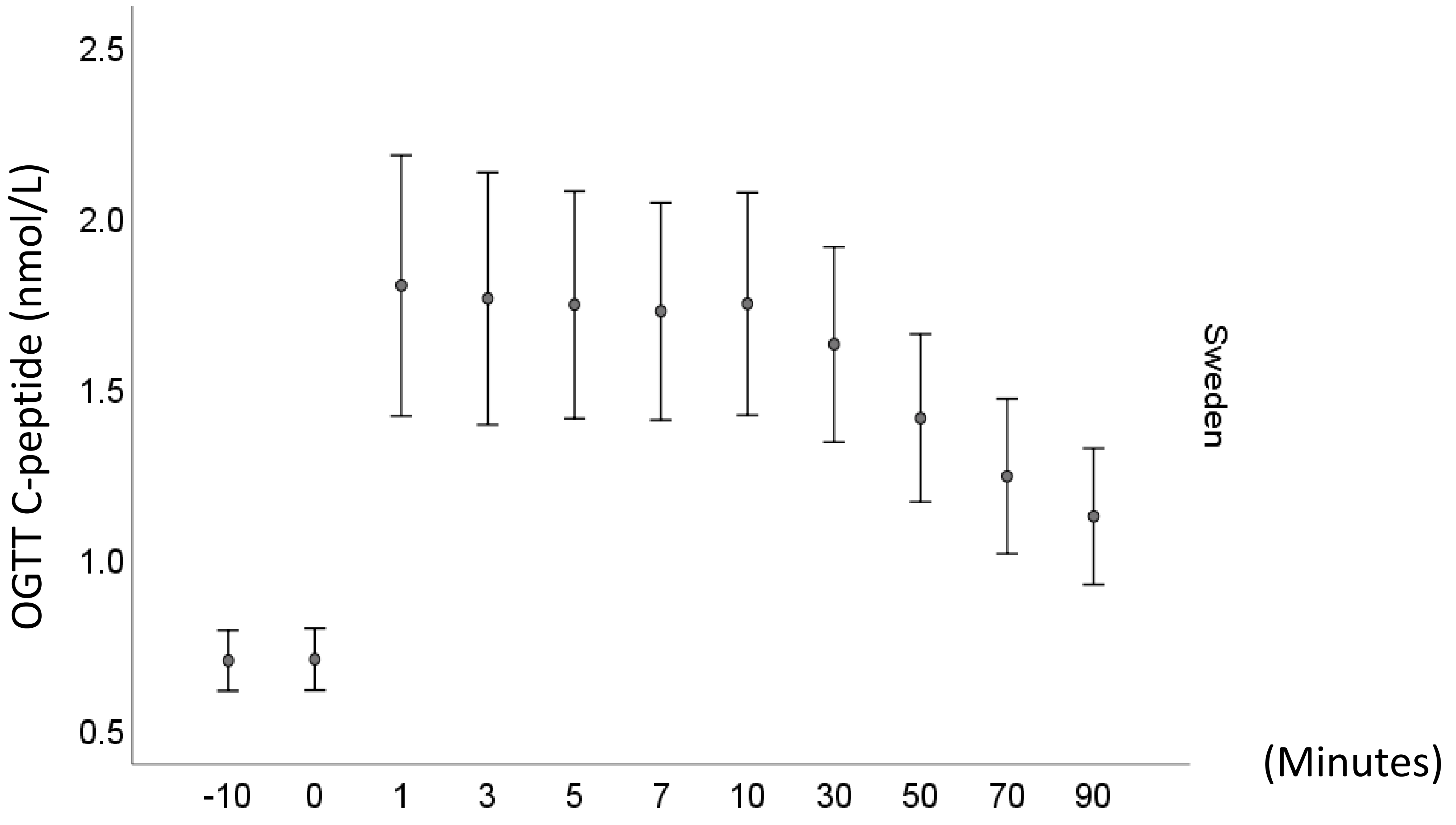
OGTT C-peptide (nmol/L)
2.5
2.0
1.5
1.0
0.5
-10
0
1
3
5
7
10
30
50
70
90
(Minutes)
Sweden
