# Supplementary material for: Beta cell function in participants with single or multiple islet autoantibodies at baseline in the TEDDY Family Prevention Study: TEFA
Source: Endocrinol Diabetes Metab. 2020 Nov 5;4(2):e00198. doi: 10.1002/edm2.198 (PMC8029501; doi:10.1002/edm2.198)
Supplement: Supplementary file 2 — Supplementary Material [file EDM2-4-e00198-s001.docx]

Supplementary Table 1. Summary of autoantibodies of subjects enrolled in the TEFA-study with a single autoantibody both in Sweden and in Finland.

|  | **Sweden** | | | | **Finland** | | | |
| --- | --- | --- | --- | --- | --- | --- | --- | --- |
| Subjects | GADA | IA2A | IAA | ZnT8(R)A | GADA | IA2A | IAA | ZnT8(R)A |
| Subject 1 | + | - | - | - |  |  |  |  |
| Subject 2 | + | - | - | - |  |  |  |  |
| Subject 3 | + | - | - | - |  |  |  |  |
| Subject 4 | - | - | + | - |  |  |  |  |
| Subject 5 | + | - | - | - |  |  |  |  |
| Subject 6 | - | - | + | - |  |  |  |  |
| Subject 7 | - | - | + | - |  |  |  |  |
| Subject 8 | - | - | + | - |  |  |  |  |
| Subject 9 | + | - | - | - |  |  |  |  |
| Subject 10 | - | - | + | - |  |  |  |  |
| Subject 11 | - | - | + | - |  |  |  |  |
| Subject 12 | - | - | + | - |  |  |  |  |
| Subject 13 | + | - | - | - |  |  |  |  |
| Subject 14 | - | - | + | - |  |  |  |  |
| Subject 15 | + | - | - | - |  |  |  |  |
| Subject 16 | + | - | - | - |  |  |  |  |
| Subject 17 | - | - | + | - |  |  |  |  |
| Subject 18 | - | - | + | - |  |  |  |  |
| Subject 19 | - | - | + | - |  |  |  |  |
| Subject 20 | + | - | - | - |  |  |  |  |
| Subject 21 | + | - | - | - |  |  |  |  |
| Subject 22 |  |  |  |  | + | - | - | - |
| Subject 23 |  |  |  |  | + | - | - | - |
| Subject 24 |  |  |  |  | + | - | - | - |
| Subject 25 |  |  |  |  | - | - | + | - |
| Subject 26 |  |  |  |  | + | - | - | - |
| Subject 27 |  |  |  |  | + | - | - | - |
| Subject 28 |  |  |  |  | - | - | + | - |
| Subject 29 |  |  |  |  | + | - | - | - |
| Subject 30 |  |  |  |  | + | + | - | - |

Supplementary Table 2. Combinations of autoantibodies of subjects enrolled in the TEFA-study with multiple autoantibodies both in Sweden and in Finland.

|  | **Sweden** | | | | **Finland** | | | |
| --- | --- | --- | --- | --- | --- | --- | --- | --- |
| Subjects | GADA | IA2A | IAA | ZnT8(R)A | GADA | IA2A | IAA | ZnT8(R)A |
| Subject 1 | + | - | + | - |  |  |  |  |
| Subject 2 | + | - | + | - |  |  |  |  |
| Subject 3 | + | - | - | + |  |  |  |  |
| Subject 4 | + | - | + | - |  |  |  |  |
| Subject 5 | + | - | + | - |  |  |  |  |
| Subject 6 | + | + | - | - |  |  |  |  |
| Subject 7 | + | - | - | + |  |  |  |  |
| Subject 8 | + | + | - | - |  |  |  |  |
| Subject 9 | + | + | - | - |  |  |  |  |
| Subject 10 | + | - | + | - |  |  |  |  |
| Subject 11 | + | + | - | - |  |  |  |  |
| Subject 12 | + | - | - | + |  |  |  |  |
| Subject 13 | + | + | - | - |  |  |  |  |
| Subject 14 | + | + | - | - |  |  |  |  |
| Subject 15 | - | + | - | + |  |  |  |  |
| Subject 16 | + | - | - | + |  |  |  |  |
| Subject 17 | + | - | - | + |  |  |  |  |
| Subject 18 | + | + | - | + |  |  |  |  |
| Subject 19 | + | - | - | + |  |  |  |  |
| Subject 20 | + | + | - | + |  |  |  |  |
| Subject 21 | + | + | - | + |  |  |  |  |
| Subject 22 | + | + | - | + |  |  |  |  |
| Subject 23 | + | - | + | + |  |  |  |  |
| Subject 24 | + | + | + | + |  |  |  |  |
| Subject 25 | + | + | + | + |  |  |  |  |
| Subject 26 | + | - | - | + |  |  |  |  |
| Subject 27 | + | + | - | + |  |  |  |  |
| Subject 28 | + | + | - | + |  |  |  |  |
| Subject 29 | + | + | + | + |  |  |  |  |
| Subject 30 | + | + | + | + |  |  |  |  |
| Subject 31 |  |  |  |  | - | + | + | - |
| Subject 32 |  |  |  |  | + | - | - | + |
| Subject 33 |  |  |  |  | + | - | - | + |
| Subject 34 |  |  |  |  | + | + | + | + |
| Subject 35 |  |  |  |  | + | - | + | - |
| Subject 36 |  |  |  |  | + | + | - | - |
| Subject 37 |  |  |  |  | + | - | + | - |
| Subject 38 |  |  |  |  | + | + | - | - |
| Subject 39 |  |  |  |  | - | + | + | - |
| Subject 40 |  |  |  |  | + | + | - | - |
| Subject 41 |  |  |  |  | + | + | - | - |
| Subject 42 |  |  |  |  | + | + | - | - |
| Subject 43 |  |  |  |  | + | + | + | - |
| Subject 44 |  |  |  |  | + | + | + | - |
| Subject 45 |  |  |  |  | + | + | + | - |
| Subject 46 |  |  |  |  | + | + | + | - |

Supplementary Table 3. Metabolic results in relation to gender and age (below 10 yrs, above 10 yrs, below 18 yrs and above 18 yrs) of subjects enrolled in the TEFA-study with a single autoantibody (AAB) (n=30) and with multiple autoantibodies (n=46) both in Sweden and in Finland. (K-value was not obtained in Finland due to the short IvGTT).

|  | **Sweden** | | | **Finland** | | |  |
| --- | --- | --- | --- | --- | --- | --- | --- |
| Variable | n (%) | Value (mean ± SD) | p | n (%) | Value (mean ± SD) | p | |
| Single AAB |  |  |  |  |  |  | |
| *HbA1c (mmol/ml)* |  |  |  |  |  |  | |
| Women | 10 (33%) | 31.8 ± 2.25 | 0.0180 | 8 (27%) | 35.6 ± 4.57 | N/A | |
| Men | 10 (33%) | 34.7 ± 2.71 |  | 1 (3%) | 35.0 ± 0.00 |  |  |
|  |  |  |  |  |  |  | |
| Multiple AAB |  |  |  |  |  |  | |
| *HbA1c (mmol/ml)* | *28 (61%)* | *34.1 ± 3.44* | *N/A* | *16 (35%)* | *34.8 ± 6.12* | *N/A* | |
| Women | 16 (35%) | 34.7 ± 4.03 | 0.282 | 8 (17%) | 35.0 ± 8.26 | 0.907 | |
| Men | 12 (26%) | 33.3 ± 2.38 |  | 8 (17%) | 34.6 ± 3.46 |  |  |
| Below 10 yrs | 5 (11%) | 34.2 ± 2.68 | 0.529 | 12 (26%) | 35.0 ± 7.10 | 0.817 | |
| Above 10 yrs | 17 (37%) | 34.1 ± 3.98 |  | 3 (6%) | 34.0 ± 1.73 |  |  |
| Below 18 yrs | 23 (50%) | 34.0 ± 3.64 | 0.428 | 15 (33%) | 34.8 ± 6.34 | N/A | |
| Above 18 yrs | 5 (11%) | 34.4 ± 2.61 |  | 1 (2%) | 35.0 ± 0.00 |  |  |
| *Vitamin D μg* | *30 (65%)* | *55.8 ± 19.4* | *N/A* | *15 (33%)* | *88.1 ± 36.0* | *N/A* | |
| Women | 16 (35%) | 56.5 ± 19.7 | 0.630 | 7 (15%) | 98.0 ± 41.9 | 0.336 | |
| Men | 14 (30%) | 53.2 ± 16.9 |  | 8 (17%) | 79.4 ± 30.0 |  |  |
| Below 10 yrs | 7 (15%) | 62.0 ± 13.1 | 0.315 | 11 (24%) | 86.9 ± 37.5 | 0.684 | |
| Above 10 yrs | 17 (37%) | 52.9 ± 21.7 |  | 3 (6%) | 97.3 ± 42.4 |  |  |
| Below 18 yrs | 25 (54%) | 55.8 ± 19.4 | 0.602 | 14 (30%) | 89.1 ± 37.1 | N/A | |
| Aabove 18 yrs | 5 (11%) | 51.0 ± 11.1 |  | 1 (2%) | 73.0 ± 0.00 |  |  |
|  |  |  |  |  |  |  | |
| *P-glucose(mmol/L) M10** |  |  |  |  |  |  | |
| Women | 16 (35%) | 5.59 ± 0.85 | 0.864 | 8 (17%) | 5.05 ± 0.50 | 0.617 | |
| Men | 14 (30%) | 5.59 ± 0.85 |  | 8 (17%) | 4.88 ± 0.83 |  |  |
| *FPIR* | *28 (61%)* | *168 ± 135* | *N/A* | *10 (22%)* | *51.8 ± 30.6* | *N/A* | |
| Women | 16 (35%) | 186 ± 170 | 0.425 | 4 (9%) | 51.8 ± 34.9 | 0.996 | |
| Men | 12 (26%) | 144 ± 86.9 |  | 6 (13%) | 51.9 ± 31.0 |  |  |
| Below 10 yrs | 6 (13%) | 117 ± 48.7 | 0.245 | 7 (15%) | 58.0 ± 32.4 | 0.458 | |
| Above 10 yrs | 16 (35%) | 196 ± 157 |  | 2 (4%) | 37.3 ± 35.7 |  |  |
| Below 18 yrs | 23 (50%) | 171 ± 137 | 0.818 | 9 (7%) | 53.4 ± 32.1 | N/A | |
| Above 18 yrs | 5 (11%) | 156 ± 140 |  | 1 (2%) | 37.9 ± 0.00 |  |  |
|  |  |  |  |  |  |  | |
| *K-value* | *30 (65%)* | *34.1 ± 3.44* | *N/A* |  |  |  | |
| Women | 16 (35%) | 2.00 ± 0.78 | 0.873 |  |  |  | |
| Men | 14 (30%) | 2.05 ± 0.71 |  |  |  |  | |
| Below 10 yrs | 7 (15%) | 2.38 ± 0.78 | 0.236 |  |  |  | |
| Above 10 yrs | 17 (37%) | 1.88 ± 0.78 |  |  |  |  | |
| Below 18 yrs | 25 (54%) | 2.02 ± 0.78 | 0.627 |  |  |  | |
| Above 18 yrs | 5 (11%) | 2.06 ± 0.46 |  |  |  |  | |

* M10 = 10 minutes prior to ingestion of glucose.

Supplementary Table 4a. Oral glucose tolerance test (OGTT) glucose results for subjects enrolled in the TEFA-study with a single autoantibody (AAB) (n=30) in Sweden and Finland. Reference values for glucose in the fasting condition: ≤6,1 mmol/L and at 120 min ≤7,8 mmol/L.

|  |  | **Sweden** |  | **Finland** |
| --- | --- | --- | --- | --- |
| Timepoints (minutes) | n (%) | Glucose (mmol/L)  Median; range | n (%) | Glucose (mmol/L)  Median; range |
| M10* | 21 (70%) | 5.30 (4.60 – 6.20) | 9 (30%) | 5.30 (4.30 – 6.80) |
| 0 | 21 (70%) | 5.20 (4.20 – 6.10) | 9 (30%) | 5.30 (4.20 – 6.90) |
| 30 | 21 (70%) | 7.50 (4.40 – 10.6) | 9 (30%) | 7.70 (6.20 – 11.3) |
| 60 | 21 (70%) | 5.90 (3.60 – 11.4) | 9 (30%) | 6.20 (3.40 – 12.7) |
| 90 | 21 (70%) | 5.30 (3.00 – 8.90) | 9 (30%) | 5.60 (3.80 – 14.2) |
| 120 | 21 (70%) | 4.90 (3.40 – 6.90) | 8 (27%) | 4.75 (3.10 – 7.70) |

* M10 = 10 minutes prior to ingestion of glucose.

Supplementary Table 4b. Oral glucose tolerance test (OGTT) insulin results for subjects enrolled in the TEFA-study with a single autoantibody (AAB) (n=30) in Sweden and Finland. Reference values for insulin in fasting condition: min <25mlU/L in Sweden and 2.6-25 mU/L in Finland.

|  |  | **Sweden** |  | **Finland** |
| --- | --- | --- | --- | --- |
| Timepoints (minutes) | n (%) | Insulin (mlU/L)  Median; range | n (%) | Insulin (mU/L)  Median; range |
| M10* | 18 (60%) | 7.50 (2.00 -20.0) | 7 (23%) | 11.4 (2.20 – 16.8) |
| 0 | 18 (60%) | 7.00 (3.00 – 23.0) | 7 (23%) | 8.40 (2.90 – 18.1) |
| 30 | 20 (67%) | 57.0 (8.00 – 216) | 7 (23%) | 75.4 (7.90 – 206) |
| 60 | 19 (63%) | 53.0 (22.0 – 317) | 7 (23%) | 62.5 (10.4 – 270) |
| 90 | 20 (67%) | 31.5 (11.0 – 206) | 7 (23%) | 67.9 (13.9 – 350) |
| 120 | 19 (63%) | 27.0 (13.0 – 143) | 6 (20%) | 78.4 (14.5 – 140) |

* M10 = 10 minutes prior to ingestion of glucose.

Supplementary Table 4c. Oral glucose tolerance test (OGTT) C-Peptide results for subjects enrolled in the TEFA-study with a single autoantibody (AAB) (n=30) in Sweden and Finland. Reference values for C-Peptide in fasting condition: 0,37-1.5 nmol/L in Sweden and in Turku and >0.9 nmol/L in Oulu.

|  |  | **Sweden** |  | **Finland** |
| --- | --- | --- | --- | --- |
| Timepoints (minutes) | n (%) | C-Peptide (nmol/L)  Median; range | n (%) | C-Peptide (nmol/L)  (Minimum-Maximum) |
| M10* | 18 (60%) | 0.69 (0.43 – 1.40) | 6 (20%) | 0.45 (0.22 – 0.56) |
| 0 | 20 (67%) | 0.65 (0.42 – 1.10) | 6 (20%) | 0.49 (0.20 – 0.63) |
| 30 | 20 (67%) | 2.15 (0.96 – 4.10) | 6 (20%) | 1.53 (0.41 – 2.72) |
| 60 | 20 (67%) | 2.35 (1.50 – 6.40) | 6 (20%) | 2.17 (0.49 – 3.18) |
| 90 | 20 (67%) | 1.90 (0.92 – 5.00) | 6 (20%) | 1.93 (0.62 – 2.71) |
| 120 | 19 (63%) | 1.80 (0.92 – 4.60) | 5 (17%) | 2.05 (0.74 – 2.73) |

* M10 = 10 minutes prior to ingestion of glucose.

Supplementary Table 5. Area under the curve (AUC) results in relation to gender and age (below 18 yrs and above 18 yrs) of subjects enrolled in the TEFA-study with a single autoantibody (AAB) (n=21) in Sweden. (AUC in relation to gender and age was not estimated in Finland due to too few cases).

| **Sweden** | | | |
| --- | --- | --- | --- |
| Variable | n (%) | Value (mean ± SD) | p |
| *AUC-Glucose* |  |  |  |
| Women | 10 (48%) | 700 ± 140 | 0.411 |
| Men | 11 (52%) | 749 ± 127 |  |
| Below 18 yrs | 2 (10%) | 755 ± 52.0 | 0.746 |
| Above 18 yrs | 19 (90%) | 722 ± 138 |  |
| *AUC-Insulin* |  |  |  |
| Women | 9 (43%) | 5118 ± 4181 | 0.779 |
| Men | 8 (38%) | 5574 ± 1794 |  |
| Below 18 yrs | 2 (10%) | 4598 ± 2874 | 0.741 |
| Above 18 yrs | 15 (71%) | 5431 ± 3314 |  |
| *AUC-C-Peptide* |  |  |  |
| Women | 10 (48%) | 232 ± 89.4 | 0.504 |
| Men | 9 (43%) | 257 ± 66.2 |  |
| Below 18 yrs | 2 (10%) | 186.90 ± 92.07 | 0.286 |
| Above 18 yrs | 17 (81%) | 250.84 ± 76.70 |  |

Supplementary Table 6. Oral glucose tolerance test (OGTT) in relation to subjects with a single autoantibody (n=21) positive for GADA in Sweden or IAA. (OGTT in relation to subject positive for GADA or IAA was not estimated in Finland due to too few cases).

|  | **Sweden** | | | |
| --- | --- | --- | --- | --- |
| Variable | n | Value (mean ± SD) | P |  |
| P-glucose M10* IAA | 11 (52%) | 5.25 ± 0.35 | 0.308 |  |
| P-glucose M10* GADA | 10 (48%) | 5.46 ± 0.57 |  |  |
| P-glucose 0 IAA | 11 (52%) | 5.32 ± 0.39 | 0.613 |  |
| P-glucose 0 GADA | 10 (48%) | 5.21 ± 0.61 |  |  |
| P-glucose 30 IAA | 11 (52%) | 7.61 ± 1.50 | 0.865 |  |
| P-glucose 30 GADA | 10 (48%) | 7.49 ± 1.64 |  |  |
| P-glucose 60 IAA | 11 (52%) | 6.55 ± 2.09 | 0.699 |  |
| P-glucose 60 GADA | 10 (48%) | 6.19 ± 1.94 |  |  |
| P-glucose 90 IAA | 11 (52%) | 4.86 ± 1.55 | 0.383 |  |
| P-glucose 90 GADA | 10 (48%) | 5.46 ± 1.41 |  |  |
| P-glucose 120 IAA | 11 (52%) | 5.01 ± 0.71 | 0.750 |  |
| P-glucose 120 GADA | 10 (48%) | 4.89 ± 1.00 |  |  |

* M10 = 10 minutes prior to ingestion of glucose.

Supplementary Table 7. Glucose metabolism in participants with a single autoantibody (AAB) (n=30) with either IAA (n=11) or GADA (n=17) both in Sweden and in Finland. The IAA group was split from median 10.17 U/mL and the GADA group was split from median 132 U/mL.

|  | **Low IAA^§^** | | | **High IAA^§^** | | |  |
| --- | --- | --- | --- | --- | --- | --- | --- |
| Variable | n | Range | n | | Range | p | |
| Age (yrs) | 6 | 32.4 - 45.7 | 5 | | 14.8 - 41.4 | 0.201 | |
| HbA1c (mmol/mol) | 5 | 27.0 - 38.0 | 5 | | 21.0 - 33.0 | 0.310 | |
| M10* glucose (mmol/L) | 6 | 4.60 - 5.90 | 5 | | 4.70 - 5.30 | 0.931 | |
| M10* C-Peptide (nmol/L) | 6 | 0.44 - 1.40 | 5 | | 0.43 - 0.89 | 0.421 | |
| M10* Insulin (mUI/L) / (mU/L) | 6 | 4.00 - 19.0 | 5 | | 4.00 - 14.0 | 0.548 | |
| IAA (U/mL) | 6 | 4.53 - 10.2 | 5 | | 22.3 - 34.1 | 0.004 | |
| 120 glucose (mmol/L) | 6 | 3.80 - 6.30 | 5 | | 4.50 - 5.30 | 0.931 | |
| 120 C-Peptide (nml/L) | 5 | 1.20 - 4.60 | 4 | | 0.92 - 1.40 | 0.286 | |
| 120 Insulin (mUI/L) / (mU/L) | 6 | 13.0 - 143 | 5 | | 15.0 - 27.0 | 0.905 | |
|  | **Low GADA^ƒ^** | | **High GADA^ƒ^** | | |  | |
| Age (yrs) | 8 | 7.10 - 47.0 | 9 | | 9.60 - 45.1 | 0.673 | |
| HbA1c (mmol/mol) | 8 | 31.0 - 38.0 | 9 | | 30.0 - 45.0 | 0.481 | |
| M10* glucose (mmol/L) | 8 | 4.30 - 5.90 | 9 | | 4.60 - 6.80 | 0.093 | |
| M10* C-Peptide (nmol/L) | 5 | 0.22 - 0.79 | 7 | | 0.27 - 1.10 | 1.00 | |
| M10* Insulin (mUI/L) / (mU/L) | 6 | 4.70 - 16.8 | 7 | | 2.00 - 20.0 | 0.628 | |
| GADA (U/mL) | 8 | 35.0 - 115 | 9 | | 132 - 1.5x10^6^ | <0.001 | |
| 120 glucose (mmol/L) | 8 | 3.10 - 6.70 | 8 | | 3.40 - 7.70 | 0.645 | |
| 120 C-Peptide (nml/L) | 8 | 0.74 - 2.30 | 9 | | 1.50 - 2.73 | 0.833 | |
| 120 Insulin (mUI/L) / (mU/L) | 8 | 14.5 - 133 | 9 | | 21.0 - 140 | 0.852 | |

*M10 = 10 minutes prior to ingestion of glucose.

^§^As IAA assays are less well standardized than GADA assays and there were 11 participants that were IAA positive in Sweden and only 2 from Finland we only tested those that were IAA positive in Sweden.

**^ƒ^**Of those 17 participants that were positive for GADA alone, 10 were from Sweden and 7 were from Finland.

Supplementary Table 8a. Intravenous glucose tolerance test (IvGTT) glucose results for subjects enrolled in the TEFA-study with multiple autoantibodies (AAB) (n=46) in Sweden and Finland. Reference values for glucose in fasting condition: ≤6,1 mmol/L and 90 min ≤7,8 mmol/L. (Due to the short IvGTT the minutes 7, 30, 50, 70 and 90 were not obtained in Finland).

|  |  | **Sweden** |  | **Finland** |
| --- | --- | --- | --- | --- |
| Timepoints (minutes) | n (%) | Glucose (mmol/L)  Median; range | n (%) | Glucose (mmol/L)  Median; range |
| M10* | 29 (63%) | 5.40 (4.00 – 7.40) | 9 (20%) | 5.05 (3.50 – 6.40) |
| 0 | 28 (61%) | 5.35 (3.90 – 21.0) | 10 (22%) | 4.95 (3.50 – 6.40) |
| 1 | 28 (61%) | 23.3 (11.1 – 27.3) | 10 (22%) | 18.9 (15.6 – 22.4) |
| 3 | 29 (63%) | 21.0 (14.5 – 25.1) | 10 (22%) | 17.2 (14.4 – 20.6) |
| 5 | 29 (63%) | 20.2 (15.9 – 25.5) | 10 (22%) | 15.7 (14.3 – 18.8) |
| 7 | 29 (63%) | 19.0 (16.7 – 23.8) |  |  |
| 10 | 29 (63%) | 17.6 (15.5 – 23.7) | 10 (22%) | 14.8 (12.8 – 18.2) |
| 30 | 29 (63%) | 10.6 (5.30 – 19.5) |  |  |
| 50 | 29 (63%) | 6.75 (3.50 – 15.9) |  |  |
| 70 | 29 (63%) | 5.60 (3.40 – 12.4) |  |  |
| 90 | 29 (63%) | 4.70 (3.50 – 10.7) |  |  |

* M10 = 10 minutes prior to ingestion of glucose.

Supplementary Table 8b. Intravenous glucose tolerance test (IvGTT) insulin results for subjects enrolled in the TEFA-study with multiple autoantibodies (AAB) (n=46) in Sweden and Finland. Reference values for insulin in fasting condition: min <25mlU/L in Sweden and 2.6-25 mU/L in Finland. (Due to the short IvGTT the minutes 7, 30, 50, 70 and 90 were not obtained in Finland).

|  |  | **Sweden** |  | **Finland** |
| --- | --- | --- | --- | --- |
| Timepoints (minutes) | n (%) | Insulin (mlU/L)  Median; range | n (%) | Insulin (mU/L)  Median; range |
| M10* | 29 (63%) | 12.0 (3.00 – 28.0) | 9 (20%) | 5.10 (1.20 – 21.9) |
| 0 | 28 (61%) | 10.5 (4.00 – 26.0) | 10 (22%) | 4.05 (1.60 – 9.50) |
| 1 | 28 (61%) | 75.0 (11.0 – 285) | 10 (22%) | 27.6 (6.00 – 53.8) |
| 3 | 29 (63%) | 62.0 (7.00 – 260) | 10 (22%) | 26.9 (4.70 – 49.8) |
| 5 | 29 (63%) | 54.0 (10.0 – 220) | 10 (22%) | 18.4 (4.60 – 37.1) |
| 7 | 29 (63%) | 50.0 (11.0 – 184) |  |  |
| 10 | 29 (63%) | 42.0 (11.0 – 710) | 10 (22%) | 16.5 (5.40 – 26.9) |
| 30 | 29 (63%) | 32.0 (6.00 – 111) |  |  |
| 50 | 29 (63%) | 21.0 (5.00 – 67.0) |  |  |
| 70 | 29 (63%) | 19.0 (4.00 – 58.0) |  |  |
| 90 | 29 (63%) | 14.0 (3.00 – 54.0) |  |  |

* M10 = 10 minutes prior to ingestion of glucose.

Supplementary Table 8c. Intravenous glucose tolerance test (IvGTT) C-Peptide results for subjects enrolled in the TEFA-study with multiple autoantibodies (AAB) (n=46) in Sweden and Finland. Reference values in fasting condition: min 0.37-1.5 nmol/L in Sweden and Turku and >0.9 nmol/L in Oulu. (Due to the short IvGTT the minutes 7, 30, 50, 70 and 90 were not obtained in Finland).

|  |  | **Sweden** |  | **Finland** |
| --- | --- | --- | --- | --- |
| Timepoints (minutes) | n (%) | C-Peptide (nmol/L)  Median; range | n (%) | C-Peptide (nmol/L)  Median; range |
| M10* | 29 (63%) | 0.67 (0.38 – 1.30) | 9 (20%) | 0.21 (0.11 – 0.38) |
| 0 | 28 (61%) | 0.64 (0.37 – 1.20) | 10 (22%) | 0.23 (0.11 – 0.50) |
| 1 | 28 (61%) | 1.60 (0.49 – 4.00) | 10 (22%) | 0.55 (0.22 – 0.85) |
| 3 | 29 (63%) | 1.70 (0.45 – 4.10) | 10 (22%) | 0.54 (0.22 – 0.86) |
| 5 | 29 (63%) | 1.80 (0.50 – 3.80) | 10 (22%) | 0.57 (0.19 – 0.86) |
| 7 | 29 (63%) | 1.70 (0.52 – 3.50) |  |  |
| 10 | 29 (63%) | 1.70 (0.55 – 3.70) | 10 (22%) | 0.61 (0.20 – 0.78) |
| 30 | 29 (63%) | 1.50 (0.66 – 3.10) |  |  |
| 50 | 29 (63%) | 1.40 (0.47 – 2.80) |  |  |
| 70 | 29 (63%) | 1.20 (0.37 – 2.70) |  |  |
| 90 | 29 (63%) | 1.00 (0.38 – 2.60) |  |  |

* M10 = 10 minutes prior to ingestion of glucose.

Supplementary Table 9. Glucose results from intravenous glucose tolerance test (IvGTT) in relation to age (below 18 yrs and above 18 yrs) of subjects enrolled in the TEFA-study with multiple autoantibodies (n=30) in Sweden. (IvGTT in relation to age was not estimated in Finland due to too few cases).

| **Sweden** | | | | | |  |
| --- | --- | --- | --- | --- | --- | --- |
| Timepoints (minutes) | n (%) | Glucose (mmol/)  Median; range | p | n (%) | Glucose (mmol/L)  Median; range | |
|  |  | *Below 18 yrs* |  |  | *Above 18 yrs* | |
| M10* | 25 (83%) | 5.50 (4.80 – 7.40) | 0.516 | 5 (17%) | 5.40 (4.00 – 6.30) | |
| 0 | 25 (83%) | 5.40 (4.60 – 21.0) | 0.300 | 5 (17%) | 5.20 (3.90 – 6.10) | |
| 1 | 25 (83%) | 23.2 (11.1 – 27.3) | 0.516 | 5 (17%) | 23.3 (20.5 – 25.8) | |
| 3 | 25 (83%) | 21.1 (14.5 – 24.9) | 0.589 | 5 (17%) | 20.8 (18.3 – 25.1) | |
| 5 | 25 (83%) | 20.2 (15.9 – 25.5) | 0.666 | 5 (17%) | 20.5 (17.8 – 23.8) | |
| 7 | 25 (83%) | 19.0 (17.1 – 23.8) | 0.552 | 5 (17%) | 19.3 (16.7 – 23.8) | |
| 10 | 25 (83%) | 17.5 (15.5 – 23.7) | 0.589 | 5 (17%) | 18.2 (15.9 – 22.7) | |
| 30 | 25 (83%) | 12.0 (8.10 – 16.5) | 0.516 | 5 (17%) | 10.4 (5.30 – 19.5) | |
| 50 | 25 (83%) | 6.60 (3.50 – 15.9) | 0.872 | 5 (17%) | 6.90 (4.60 – 10.1) | |
| 70 | 25 (83%) | 5.60 (3.40 – 12.4) | 0.914 | 5 (17%) | 4.60 (3.50 – 7.00) | |
| 90 | 24 (80%) | 4.70 (3.70 – 10.7) | 0.323 | 5 (17%) | 4.40 (3.50 -5.50) | |

* M10 = 10 minutes prior to ingestion of glucose.

**TEFA Study Acknowledgements**

The TEFA Study Group (See appendix)

Funded by Juvenile Diabetes Research Foundation (JDRF).

***Appendix***

**The TEFA Study Group**

**Sweden Clinical Center:** Daniel Agardh M.D., Ph.D. ^1^, Maria Ask, Rasmus Bennet, Henrik Borg M.D., Ph.D. ^1^, Jenny Bremer, Corrado Cilio M.D., Ph.D. ^1^, Magdalena Delikat-Kulinski, Emilie Ericson-Hallström, Lina Fransson, Thomas Gard, Joanna Gerardsson, Monica Hansen, Susanne Hyberg, Fredrik Johansen, Berglind Jonasdottir M.D., Ph.D. ^1^, Silvija Jovic, Ida Jönsson, Ulla-Marie Karlsson, Anastasia Katsarou M.D., Ph.D. ^1^, Jaakko J Koskenniemi Ph.D^1^, Helena Larsson M.D., Ph.D., CO-PI, Åke Lernmark Ph.D., PI, Marielle Lindström, Markus Lundgren M.D., Ph.D. ^1^, Maria Markan, Marlena Maziarz^1^, Jessica Melin, Zeliha Mestan, Maria Månsson-Martinez^2^, Lena Nilsson, Karin Ottosson, Anita Ramelius, Kobra Rahmati, Falastin Salami M.D., Ph.D.^1^, Sara Sibthorpe, Annette Sjöberg, Birgitta Sjöberg^2^, Evelyn Tekum-Amboh, Carina Törn Ph.D. ^1^, Ulrika Ulvenhag, Anne Wallin, Åsa Wimar, Sofie Åberg.

**Finland Clinical Center, Turku:** Annika Adamsson, Jari Hakalax, Sanna Jokipuu, Jenni kaarina Rouhiainen, Tiina Johanna Kallio, Leena Karlsson, Jukka Kero M.D, Ph.D.^1^, Jakko Koskenniemi M.D, Ph.D.^1^, Laura Leppänen, Maria Leppänen, Elina Mäntymäki, Zhian Othmani, Petra Rajala, Mika Riikonen, Eija Riski, Minna Romo, Satu Ruohonen, Maija Sjöberg, Jorma Toppari M.D., Ph.D. ^1^, Sini Vainionpää, Eeva Varjonen.

**Finland Clinical Center, Oulu:** Sirpa Anttila, Tuula Arkkola, Henna Holappa, Anni Ikonen, Tea Joensuu, Minna-Liisa Koivikko, Marika Korpela, Miia Kähönen, Tiina Latva-aho, Jaana Liimatainen, Katja Multasuo, Teija Mykkänen, Olli Okkonen, Paula Ollikainen, Riitta Päkkilä, Joona Rahko, Päivi Salmijärvi, Aino Stenius, Hanne Suorsa-Routiainen, Päivi Tossavainen M.D, Ph.D.^1^, Riitta Veijola M.D., Ph.D. ^1^, Irene Viinikangas.

^1^ Co-investigator, ^2^Study Co-ordinator
